# The Relevance of Autophagy within Inner Ear in Baseline Conditions and Tinnitus-Related Syndromes

**DOI:** 10.3390/ijms242316664

**Published:** 2023-11-23

**Authors:** Gloria Lazzeri, Francesca Biagioni, Michela Ferrucci, Stefano Puglisi-Allegra, Paola Lenzi, Carla Letizia Busceti, Francesco Giannessi, Francesco Fornai

**Affiliations:** 1 Human Anatomy, Department of Translational Research and New Technologies in Medicine and Surgery, University of Pisa, 56126 Pisa, PI, Italy; gloria.lazzeri@unipi.it (G.L.); michela.ferrucci@unipi.it (M.F.); paola.lenzi@unipi.it (P.L.); giannessifrancesco@gmail.com (F.G.); 2 IRCCS, Istituto di Ricovero e Cura a Carattere Scientifico, Neuromed, 86077 Pozzilli, IS, Italy; francesca.biagioni@neuromed.it (F.B.); stefano.puglisiallegra@neuromed.it (S.P.-A.); carla.busceti@neuromed.it (C.L.B.)

**Keywords:** stereocilia, outher hair cells, inner hair cells, tectorial membrane, Hensen’s cells, reticular lamina, basilar membrane, radial shearing, hypoacusia, neurodegeneration

## Abstract

Tinnitus is the perception of noise in the absence of acoustic stimulation (phantom noise). In most patients suffering from chronic peripheral tinnitus, an alteration of outer hair cells (OHC) starting from the stereocilia (SC) occurs. This is common following ototoxic drugs, sound-induced ototoxicity, and acoustic degeneration. In all these conditions, altered coupling between the tectorial membrane (TM) and OHC SC is described. The present review analyzes the complex interactions involving OHC and TM. These need to be clarified to understand which mechanisms may underlie the onset of tinnitus and why the neuropathology of chronic degenerative tinnitus is similar, independent of early triggers. In fact, the fine neuropathology of tinnitus features altered mechanisms of mechanic-electrical transduction (MET) at the level of OHC SC. The appropriate coupling between OHC SC and TM strongly depends on autophagy. The involvement of autophagy may encompass degenerative and genetic tinnitus, as well as ototoxic drugs and acoustic trauma. Defective autophagy explains mitochondrial alterations and altered protein handling within OHC and TM. This is relevant for developing novel treatments that stimulate autophagy without carrying the burden of severe side effects. Specific phytochemicals, such as curcumin and berberin, acting as autophagy activators, may mitigate the neuropathology of tinnitus.

## 1. General Introduction

**Figure 1:**
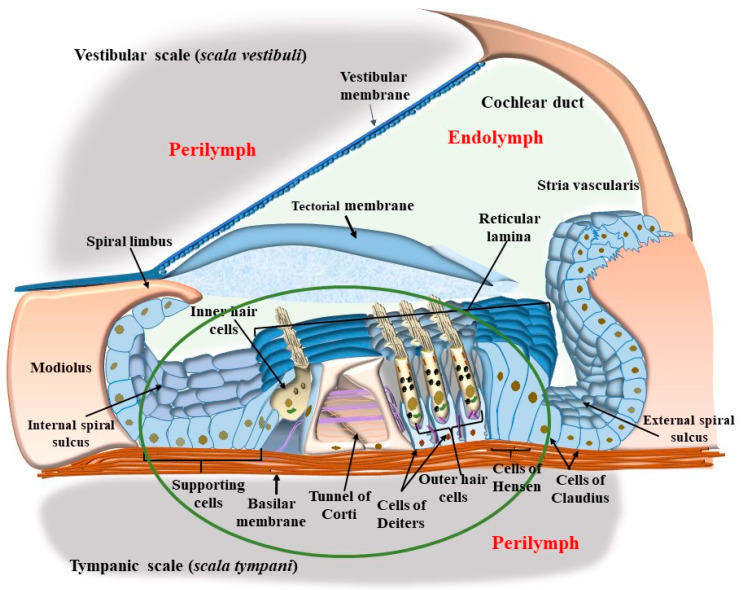
The cochlear duct and the organ of Corti. The cochlear duct is a membraneous structure that is contained within the cochlear canal. The cochlear duct contains endolymph, which is a liquid with a high concentration of cations. In the cochlear duct, the presence of endolymph is limited by the vestibular membrane and the reticular lamina (RL), where the cell membranes are sealed by Zonula Occludens 1 (ZO1)-rich gap junctions. The RL pivots at the level of supporting cells named inner pillar cells, and it is bound to an overlying membrane named the tectorial membrane (TM), which has a different pivot at the level of the spiral limbus. The different pivots and the different structures of the RL and TM produce a discrepancy during sound-induced tilting of these membranes. In fact, the RL is more flexible and moves differently from the TM. The RL is composed of the distal segment of supporting cells and classic receptor cells, so-called hair cells (HC). Hair cells are distinguished as inner HC (IHC) and outer HC (OHC) depending on their placement at the level of a central spiral cavity named the tunnel of Corti, which is limited by inner and outer pillar cells. Pillar cells, along with Deiters’ cells, Hensen’s cells, and Claudius’ cells, represent supporting cells. These cells have an intimate relationship with IHC and mostly OHC. Supporting cells are stratified on a fibrous membrane, the so-called basilar membrane (BM), which, by definition, separates the cochlear duct from the scala tympani. Indeed, the real separation between the perilymph of the scala tympani and the endolymph of the cochlear duct occurs at the level of the RL, which represents the seminal tilting structure in the Organ of Corti. The complex of BM, RL, IHC, OHC, supporting cells, and the overlying TM represent the organ of Corti (green-circled), where sophisticated mechanisms generate sound perception.

As shown in the pioneering work by Von Bekesy (1956), mammalian hearing depends on sound-evoked stimulation of the inner ear within the cochlea, which is a spiral canal placed within the petrosal division of the temporal bone (the name cochlea comes from the Greek word ‘kokhliās,’ which indicates the spiral shell of a snail).” [The eclectic life of Georg von Békésy led him to the successful paradox of starting his work in the research laboratory of the Hungarian Post Office, where he was involved in improving long-distance telephone transmission, to move within the academic environment at the University of Budapest to study the structure of the human auditory organ. In fact, the concept of acoustic transmission was so fascinating to him that he was strongly motivated to comprehend the nature of acoustic perception in the inner ear. This is why he was soon involved in autopsies focused on the petrosal part of the temporal bone while still working the rest of the day at the post office. After this early period, immediately after World War II, in 1946, he left Hungary to move to Stockholm, Sweden, where he worked at the Technical Institute and was guested at Karolinska Institutet. After a time interval, in 1947, he moved to Harvard University to embrace his passionate love for the inner ear at the psycho-acoustic laboratory. During his relevant studies condensed in the period from the 1940s to the 1960s [1], Georg von Békésy clarified the relevance of vibrations of cochlear membranes, revisiting the studies of Von Helmoltz, and he measured variations in electrical and size features of auditory receptor cells]. In fact, this canal appears as a spiral cavity carved within a dense bone. The cochlear canal is filled with a liquid named perilymph and contains a membranous spiral duct named “the cochlear duct,” which contains endolymph, as shown in representative Figure 1.

**Figure 2:**
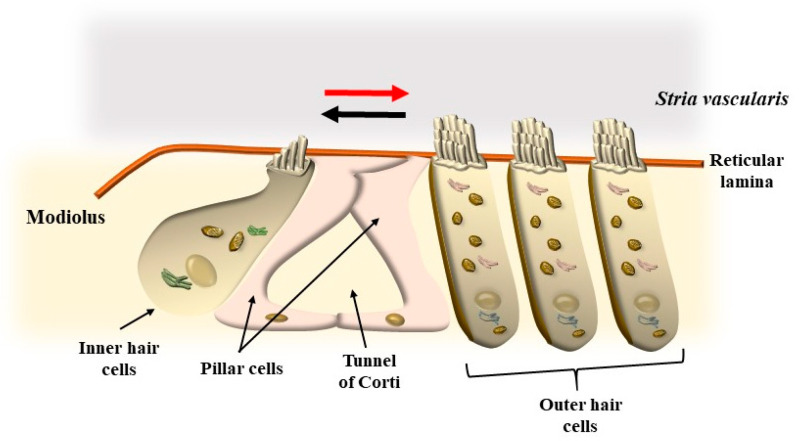
The stereocilia of HC. Both IHC and OHC possess a rigid apical apparatus, which is made of stereocilia, which possess a rigid structure anchored to the HC through an actin mesh. The length of each cilium is highly polarized since the shorter cilia in each cell are medial while the longest are lateral in the organ of Corti. Such a specific and constant polarization produces a coordinated movement of cilia within IHC and OHC, where a drive produces excitation of HC (red arrow) while the latero-medial drive generates inhibition (black arrow).

The limit between the endolymph and perilymph is marked by two membranous structures: the vestibular membrane and the tympanic membrane (basilar membrane) (Figure 1). These membranes separate the membranous duct from the other part of the cochlear canal (the vestibular scale, *scala vestibuli*, and tympanic scale, *scala tympani*, respectively, Figure 1). Indeed, the tympanic membrane, which is also known as the basilar membrane (BM), is not the real border between tympanic perilymph and ductal endolymph since the perilymph passes through the fibers and cells of the BM up to the apical region known as the reticular lamina (RL), where a ceiling between cells provides the real separation between tympanic perilymph and cochlear endolymph (Figure 1). The BM, including its fibers, nerve endings, and auditory and supporting cells underlying the tectorial membrane (TM), represents a highly connected anatomical spot named “the organ of Corti” (Figure 1). Within the organ of Corti, sounds are transduced and amplified (green-circled in Figure 1). In the organ of Corti, a number of cell types can be described, which are roughly classified either as supporting or auditory cells. Auditory cells are named “hair cells” (HC) for the presence of a bush of rigid stereocilia (SC), which are implanted on their apical membrane (Figure 1 and Figure 2).

**Figure 3:**
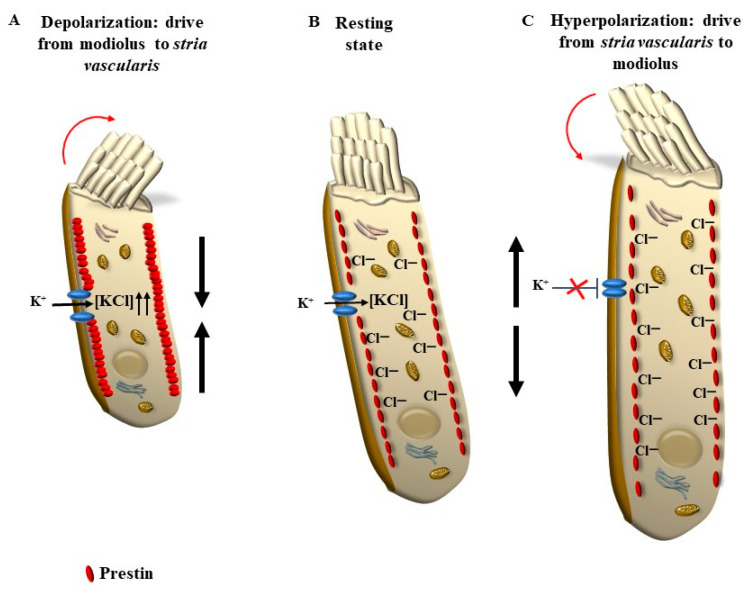
Excitation of OHC reduces cell length, while inhibition increases cell length. When SC deflection occurs in the mediolateral direction ((**A**), from modiolus to *stria vascularis*), a massive cation influx in the OHC takes place, which removes anions from the lateral protein named prestin. Such a removal shortens prestin length, which causes cell shortening. In the resting state (**B**), the entry of cations is moderate, and the length of prestin is proportionally increased. In the inhibition phase, when SC deflection occurs in the latero-medial direction (from *stria vascularis* to modiolus, (**C**)), a massive cation efflux from OHC takes place, which replaces anions binding to the protein prestin. Such a binding increases prestin length, which causes cell lengthening.

The placement of SC in each HC and within the whole Corti’s organ occurs according to a specific polarity, where the shortest SC is placed medially, towards the modiolus (the bony axis of the cochlear canal), while the longest SC is positioned laterally, towards the *stria vascularis*, which is placed on the lateral (external) side of the cochlear duct (Figure 1 and Figure 2). When the sound wave impacts the HC SC, opposite events occur depending on the direction of the mechanical impact. In detail, when the compressive phase impacts the BM coming from the *scala tympani*, a shearing of SC occurs from the modiolus to the *stria vascularis* (from the shortest to the longest SC). This mechanical stretch opens cation (K^+^ and Ca^++^) channels, which in turn generate a marked depolarization of HC (Figure 2 and Figure 3). Contrary to popular belief, when the mechanical drive pushes over the vestibular membrane, the driving polarity is directed from the *stria vascularis* towards the modiolus, which closes these cation channels and generates a marked hyperpolarization of HC (Figure 2 and Figure 3).

**Figure 4:**
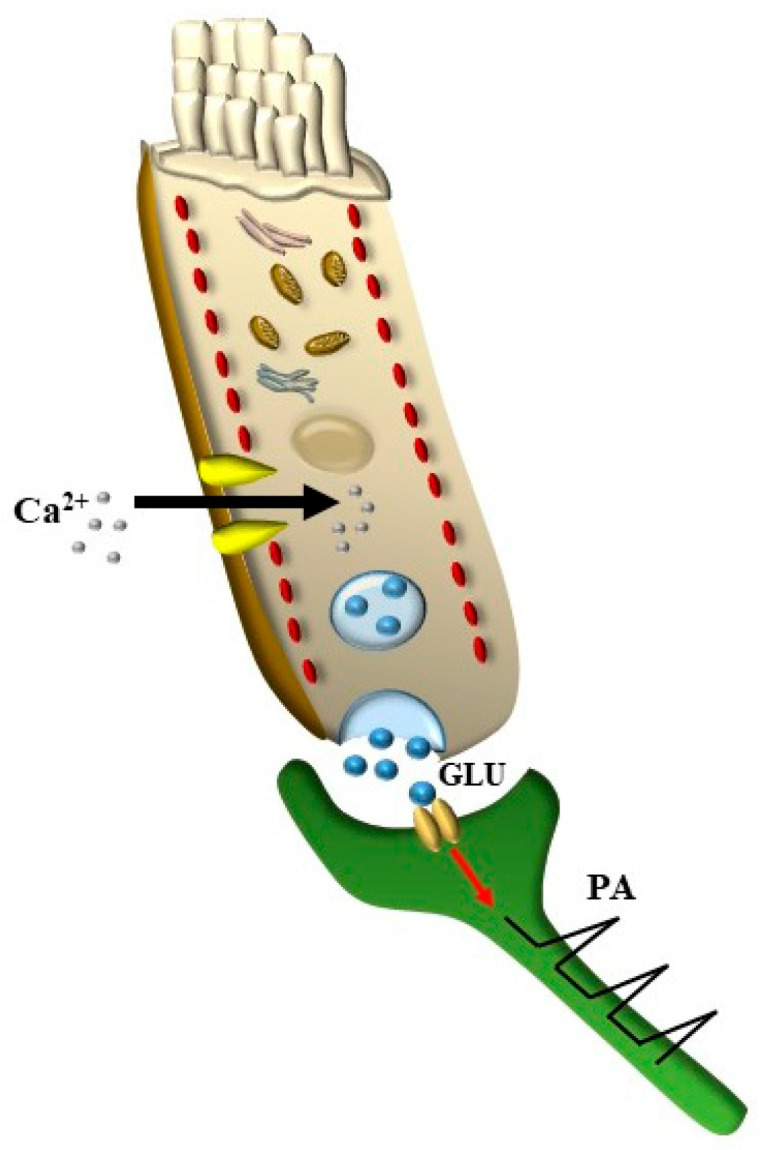
Cell excitation produces neurotransmitter release, which induces a chemical response in the acoustic nerve. When HC is excited, cation influx involves Ca^++^ ions, which, along with specific presynaptic proteins, produce the release of the neurotransmitter glutamate, which in turn excites afferent nerve endings. Such a process occurs both within OHC and IHC, although the impact on action potential in the cochlear nerve is mostly due (roughly 95%) to glutamate release from IHC.

HC depolarization is mainly generated by the inward current of K^+^, which fosters the entry of Ca^++^ ions, working in combination with specific synaptic proteins to sustain glutamate release, which triggers action potentials along the cochlear nerve (Figure 3 and Figure 4).

Two kinds of HCs are present. They are named depending on their placement, which may be inside (inner hair cells, IHC) or outside (outer hair cells, OHC) the tunnel of Corti (a spiral tunnel between inner and outer supporting pillar cells, Figure 1 and Figure 2). The tunnel of Corti extends for the whole length of the cochlear duct along its longitudinal spiral axis from the basal to the apical turn of the cochlea up to the *elicotrema*, where *scala tympani* passes into *scala vestibuli*. There are relevant differences between IHC and OHC since the activity of IHC is responsible for most action potentials in the cochlear nerve, while OHC mainly enables the activity of IHC by promoting fine-tuning and amplification of sounds.

In addition, OHC, differing from IHC, possesses the unique property of reducing or increasing cell length depending on depolarizing or hyperpolarizing currents, respectively. This is due to the unique presence within the OHC of the protein prestin (Figure 3). In this way, when OHC SCs are properly sheared and deflected from the shortest to the longest, a depolarization of OHC occurs (Figure 2). The massive entry of cations within OHC removes Cl^-^ and other anions from their binding sites within the prestin protein (Figure 2). The prestin protein is placed along the lateral domain of OHC. The removal of anions from prestin alters protein conformation, which leads to protein contraction and cell shortening (Figure 3). Conversely, when a deflection of SC takes place from the longest to the shortest, K^+^ channels are closed, which induces a full binding of anions such as Cl^-^ to prestin, leading to protein elongation and an increase in cell length (Figure 2 and Figure 3). Thus, when a sound enters the cochlea, the pressure in the vestibular scale generates cell lengthening, while the pressure in the tympanic scale produces cell shortening. In this way, cyclic motility of OHC is generated, which enhances the activity of IHC, which cyclically triggers action potentials along the cochlear nerve. Somatic electromotility of OHC emerges at P7, early in the basal turn of the cochlea, to extend later on, by P12, to the apex. The response amplitude continues to increase until P13–P14, when mature amplitudes are reached [2,3]. According to sound frequency, conformational changes in prestin lead to cycles of contraction (depolarization)/elongation (hyperpolarization) of OHC, which may reach a frequency of a thousand times per second, thus amplifying and tuning specific sound frequencies [4,5,6]. In this way, stimulation of IHC is produced according to a specific tonotopic pattern, which leads to action potentials (Figure 4). These propagate within a few specific axons of the cochlear nerve to bring selective auditory stimulations to the central nervous system (CNS), where contrast enhancement produces highly sensitive, tonotopic sound perception. These peripheral mechanisms, which take place at the level of the inner ear, are greatly sophisticated, and a complex synergism leads to the physiological stimulation of key biological structures by the physical stimulus represented by sound waves. The present review is aimed at analyzing the molecular mechanisms of sound perception to understand why alteration of metabolic pathways, such as autophagy, is involved in physiological sound perception and to comprehend the neurobiology of tinnitus. Tinnitus often anticipates and accompanies the loss of perception of sound in the presence of environmental sound stimulation (hypoacusia). In fact, in most patients complaining of tinnitus, hypoacusia is also present. Moreover, since mechanisms of HC stimulation are similar within the cochlear and vestibular systems, a number of syndromes exist where auditory alterations are concomitant with vestibular symptoms such as vertigo and loss of balance. Therefore, a glimpse of concomitant vestibular alterations will be provided in the course of the manuscript.

The major focus of the present review consists of dissecting those specific molecular events, with a focus on autophagy, which may lead to tinnitus as a potential target for phytochemicals. Therefore, physiological mechanisms leading to cochlear perceptions need to be reviewed since their alterations under autophagy dysfunction lead to tinnitus, hypoacusia, and sometimes vestibular symptoms. These mechanisms are highly sophisticated, and their knowledge constantly increases due to intense research efforts. Therefore, in the first part of the review, we are summarizing updated concepts about physiological stimulation of the inner ear in order to specifically identify those structures and events that may lead to tinnitus and hypoacusia while being targets for phytochemical-induced symptom restoration.

## 2. A Definition of Tinnitus as Altered Mechanisms of Auditory Stimulation

**Figure 5:**
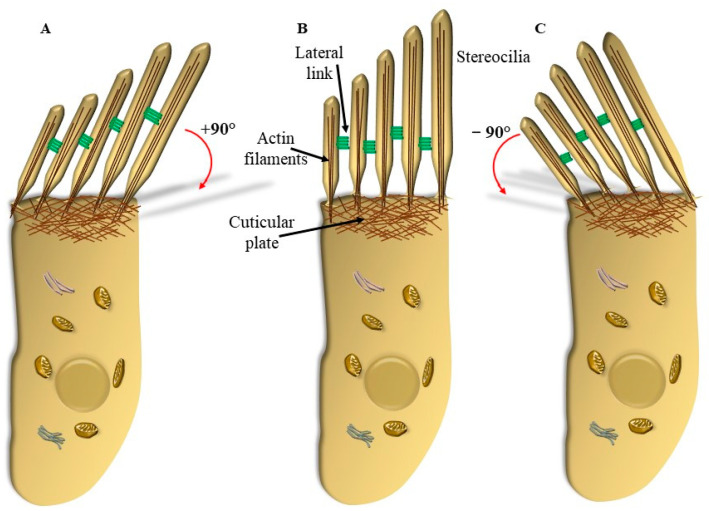
The movement of each stereocilium affects the lateral and apical membranes of the contiguous stereocilia (SC). The package of SC implanted at the apical surface of HC is connected by lateral ankle links, which lead to a concomitant movement of the whole package of SC. These lateral links are shown in green and connect the shortest and longest SC in a way that leads to the same polarization of the whole package. In this way, there is a similar movement both during excitation and inhibition, which produces an angular deflection ranging from −90° to +90° between the long axis of the cilium and the apical membrane of the HC. In detail, when SC undergoes mediolateral deflection (**A**), a depolarization occurs compared with resting conditions (**B**), when SC is orthogonal to the apical membrane and the cell is slightly depolarized. When SC is deflected from the longest to the shortest, hyperpolarization occurs (**C**). Such a movement of SC, which is induced by sound waves, is concomitant with the altered conformation of the basal insert of each stereocilium in the apical membrane of HC. Here, a cuticular plate is present, which is produced by a dense actin mesh, where actin filaments running along the axis of the stereocilium are included in the dense cytoskeleton present in the apical domain of HC.

Tinnitus is the occurrence of auditory perceptions in the absence of external sound stimuli. A number of cases of tinnitus occur during degeneration of the peripheral auditory system and/or abnormalities within the central auditory pathways, nuclei, and auditory cortex. A trivial approach distinguishes between peripheral tinnitus, which is expected to involve cochlear auditory cells and/or cochlear nerve, and central tinnitus, which is supposed to be produced on central auditory pathways and centers. Indeed, when abnormal stimulation of OHC and IHC occurs, a reflection within the central auditory pathways, nuclei, and cortex eventually occurs. These plastic phenomena lead to erasing a rigid nosographic classification between peripheral and central tinnitus since all structures involved are contaminated by abnormal sound transduction. Tinnitus originates from a dysfunction of auditory cells (hair cells) and their relationship with the TM within the Corti’s organ within the cochlear duct (Figure 1). As briefly mentioned, two kinds of auditory HC are present in the Corti’s organ, IHC and OHC, depending on their placement inside or outside, respectively, of the tunnel of Corti (Figure 1 and Figure 2). The physiological roles of IHC and OHC are different, despite their quite similar structures. Thus, IHCs are the main source of fibers for the cochlear nerve and convey to the CNS the main stimulus for auditory perception, while OHC serves mainly as an amplifier and a fine tuner to support the sensitivity and specificity for IHC stimulation, despite providing some fibers to the cochlear nerve. Both IHC and OHC are gifted with SC (hence their name, “hair cells”). As mentioned, the shorter cilia are placed closer to the modiolus, and the longest cilia are closer to the *stria terminalis* (Figure 2). Stereocilia stand as rigid structures anchored by a thinner rootlet to the apical membrane of the HC [7]. In fact, SCs are attached to the distal membrane of HC through an actin mesh named the cuticular plate (Figure 5). 

**Figure 6:**
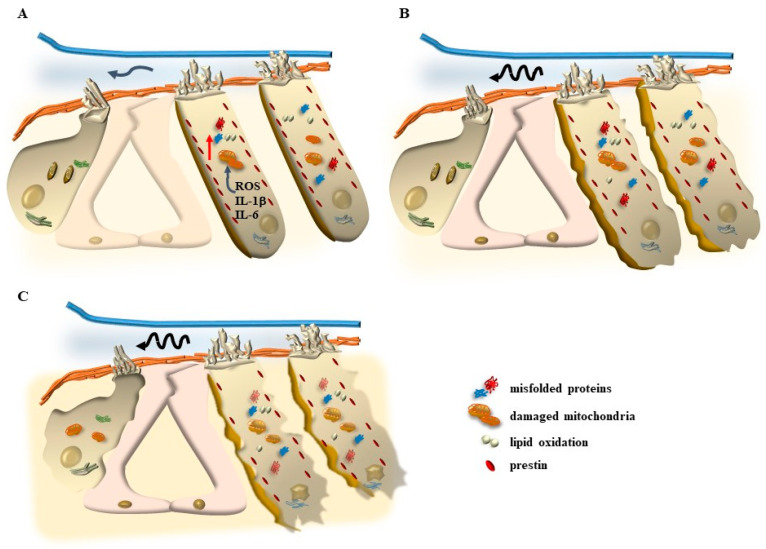
Early, progressive alterations of HC characterize tinnitus-related neurodegeneration. In the course of degenerative phenomena occurring in chronic tinnitus, there is a progressive loss of specific structural features of HC. Specifically, (**A**) at early stages, there is a selective rupture of OHC SC in the absence of any alteration at the level of IHC. This early morphological effect is considered to be concomitant with a specific functional defect in mechano-electrical transduction (MET), which is the mechano-electrical signaling occurring at the level of OHC SC. These phenomena are generated by a number of molecular mechanisms, including reactive oxygen species (ROS), altered mitochondria, inflammatory cytokines, and the accumulation of misfolded proteins, which are mostly bound to defective autophagy. At later stages, the pathology of SC extends to the entire OHC, which is affected in the whole cell volume (**B**). Later on, IHC SC started to be altered as well (**C**). At this stage, some OHC disappears, and there is a marked reduction in acoustic activity (presbyacusis). During the progression of HC degeneration, when IHCs are also involved, OHC may disappear. A derangement of OHC SC, which is already evident at early stages (**A**), may impair the ability of sound to produce an effective and appropriate stimulation of OHC, and hence even the activity of IHC may be reverted. It is remarkable that despite a number of protein alterations and lipid peroxidation along with mitochondrial defects taking place within OHC, the amount of the prestin protein is slightly affected, being actually less abundant.

This apparatus supports the placement of each cilium. Thus, all SC in each HC have remarkable rigidity, and they move as a whole by reducing the insert angle between the cilia and the apical membrane of the HC from −90° to 0° and up to +90° (Figure 5). The cuticular plate is a dense filamentous actin mesh where actin filaments in the SC merge and connect with an anchoring apparatus at the apical cell membrane. In this way, when SCs are working properly, they deflect on the cuticular plate as a whole rigid stick rather than bending their vertical shape (Figure 5). These orchestrated movements are enabled by side (lateral, ankle) links, which mechanically connect the package of SC within each HC (Figure 5). It is remarkable that, in most cases of peripheral tinnitus, the process starts with a selective degeneration of OHC, which appears to be damaged even when IHCs are fairly preserved. When the disease progresses, a clear pathology extends to IHC, although OHC remains more affected than IHC, and they may completely disappear (Figure 6). Remarkably, such a progression, which is clearly defined during the slow process of degenerative tinnitus, is mimicked by all kinds of persistent tinnitus (following ototoxic drugs or following repeated exposure to loud noise). In most of these cases, tinnitus appears along with progressive hearing loss (presbyacusia or presbyacusis [8]).

Thus, peripheral tinnitus is mostly due to a degeneration of peripheral structures within the inner ear, starting with OHC. This type of tinnitus, at present, lacks standardized, effective remedies. There are a number of generic conditions that predispose to the onset of peripheral tinnitus, including loud noise, ototoxic chemicals, and age-related or inherited degenerative disorders. Most of these factors per se do not tell much about the molecular mechanisms that generate tinnitus. It is likely that, in most cases, these various factors may converge upon a few molecular mechanisms that damage OHC, starting with SC. In fact, most cases of tinnitus are due to early dysfunction of the OHC SC, which progresses towards degeneration of the whole OHC, which is responsible for hearing loss (Figure 6). Thus, in most cases, when tinnitus appears, it is likely to be produced by inappropriate activity of OHC SC within the inner ear, which eventually extends to central auditory pathways (thereby leading to combined peripheral and central tinnitus).

It is now established that tinnitus is generated by early molecular dysfunctions and subsequent cytopathology of auditory HC, which are placed within the Corti’s organ of the cochlea (Figure 6). This kind of tinnitus is mostly associated with age and persists chronically, leading to a loss of hearing (presbyacusia or presbyacusis). In fact, when analyzing people affected by tinnitus, more than 63% of patients complain of hearing loss. Nonetheless, as we shall see, even when tinnitus is triggered by repeated loud noise exposure, ototoxic drugs, some age-related phenomena, or inherited disorders, the subcellular alterations recapitulate those occurring during degenerative tinnitus, and there is a remarkable overlapping between the molecular pathways being affected. This allows us to analyze degenerative tinnitus as a phenomenon that progresses similarly to all kinds of peripheral tinnitus. The occurrence of convergent alterations in key molecular pathways of tinnitus explains why potential remedies are effective for peripheral tinnitus generated by diverse factors. Roughly three-quarters of patients suffering from degenerative tinnitus possess neural hearing loss, which is mostly due to an impairment of OHC. Therefore, to comprehend how tinnitus is generated, an updated overview of key events occurring during the physiological activity of OHC needs to be added.

## 3. Specific Activities of Outer Hair Cells

As reported, a remarkable difference between OHC and IHC concerns the occurrence of the protein prestin, which is key to modifying cell length; prestin selectively occurs within OHC (Figure 3).

The presence of prestin is relevant to sustain the key role of OHC in tuning and amplifying IHC activity and makes OHC a sophisticated system in tuning and amplifying the process of sound perception [9,10]. The high vulnerability to degeneration makes OHC the crucial spot to understand the neurobiology of tinnitus. According to comprehensive manuscripts [11,12], the specific activity of OHC provides three kinds of signaling.

### 3.1. Mechano-Electrical Signaling (or Transduction, MET)

**Figure 7:**
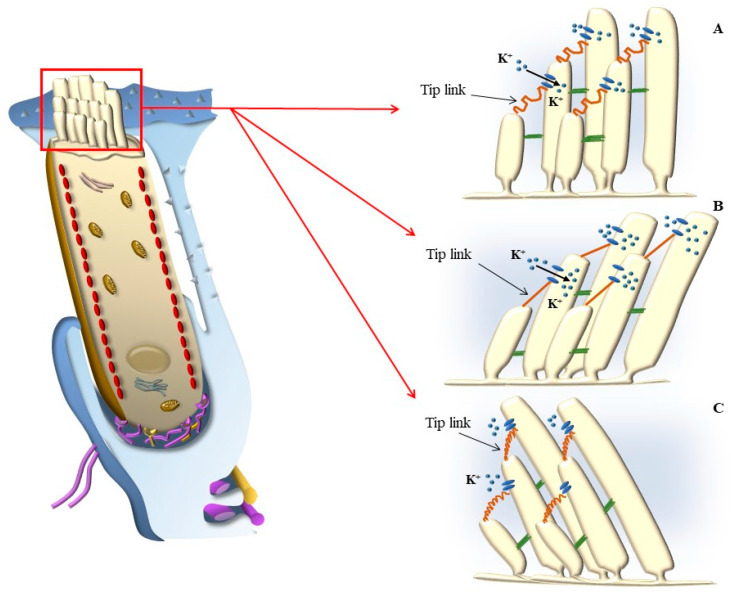
The role of tip links. In resting conditions (**A**), the position of the SC is orthogonal to the apical membrane of the HC. In this condition, the tip links, which connect the apex to the lateral membrane of contiguous SC, keep a slight tension, which partially keeps protein comformation within cation channels, which is able to produce only partial channel opening. This allows the entry of some cations within HC. During excitation (**B**), the coordinated movements of SC within the package of each HC, which are granted by lateral links, generate a gradient of tension at the level of the tip links, which connect the apex of a cilium to the lateral membrane of the nearest SC. During excitation, the movement is directed towards the longest SC, and the tension of the tip links increases, leading to an altered conformation of cation channels that are more likely to be opened, thus allowing the entry of cations within HC. Contrary to popular belief, when a coordinated movement of SC proceeds from the longest to the shortest SC (**C**), the tension of the tip links is reduced, and the cation channels are no longer mechanically stimulated, which leads to closing the entry of cations.

This is produced at the level of the lateral membrane of OHC SC, where it is generated by the mechanic stretch of the tip link binding the tip of a cilium with the side membrane of the closest longer cilium within the hair bundles (Figure 7). Such a mechanical stretch opens K^+^ channels [13], which generate an inward cation current; in this way, a mechanical stimulus generates an electrical effect.

### 3.2. Electro-Mechanical Signaling

This is produced along the lateral membrane of OHC, where the inward current of K^+^ ions removes anions, mostly Cl^−^, from the protein prestin (Figure 3). Such a removal involves anions responsible for prestin conformational changes [14]. This produces a conformational change leading to a decrease in prestin protein length, which is proportional to anion removal. Contrary to popular belief, when hyperpolarization occurs, anions bind prestin, which is proportionally elongated (Figure 3). These electrically induced conformational changes in prestin within OHC determine the mechanical shortening or lengthening of a single SC, and these length variations extend to the whole OHC (Figure 3). Thus, along the lateral membrane of OHC, an electrical alteration generates a mechanical effect. 

### 3.3. Electro-Chemical Signaling

**Figure 8:**
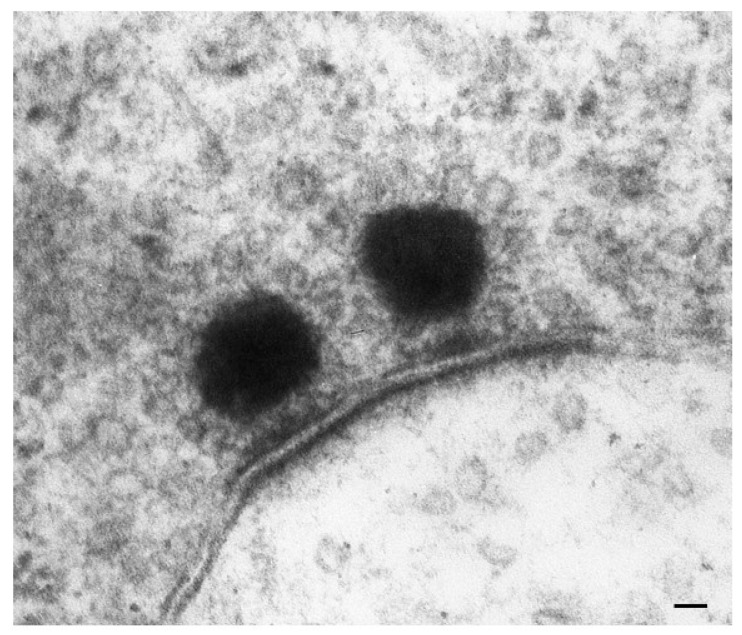
Representative micrograph of two synaptic bodies within HC. Within HC, neurotransmitter release occurs according to specific pre-synaptic structures named synaptic bodies. Two synaptic bodies appear as electron-dense structures that are placed in close relationship with the plasma membrane of HC. The synaptic body generates a number of vesicles with high variability in size, some of which are very small while others are very large. Such a release apparatus allows *quasi-*analogic neurochemical signaling, which is proportional to electrotonic excitation. This allows adjustment gradually to neurotransmitter release with the amount of excitation/inhibition occurring within HC. The original unpublished micrograph was obtained with osmium contrast enhancement. Scale bar = 7 nm.

**Figure 9:**
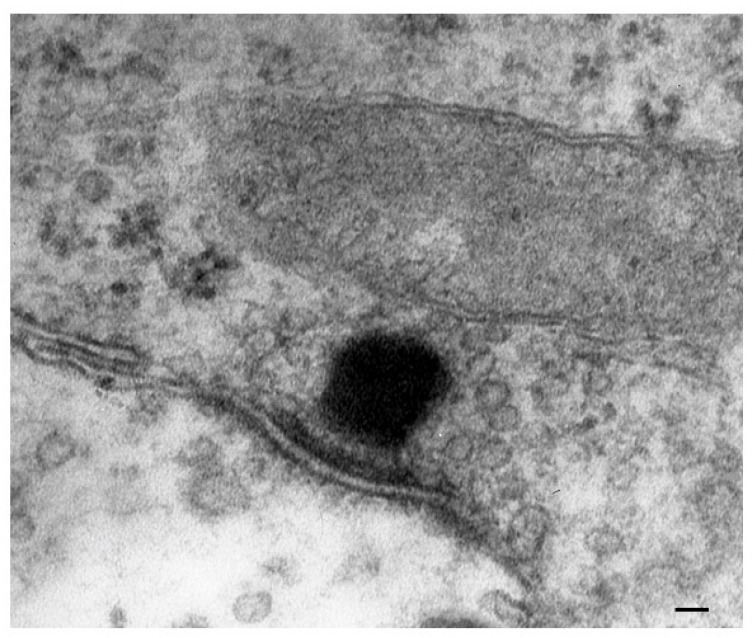
Representative micrograph of mitochondrial placement close to synaptic bodies within HC. The process of neurotransmitter release occurs at a high energy cost. This is why the occurrence of mitochondria is key to the proximity of synaptic bodies. In this representative picture at transmission electron microscopy, a mitochondrion is evident over a synaptic body, which is surrounded by different-sized vesicles. The original, unpublished micrograph was obtained with osmium contrast enhancement. Scale bar = 7 nm.

This occurs at the level of the basal membrane of HC, where the currents generated by inward/outward movements of K^+^ ions open or close, respectively, Ca^++^ channels and modulate Ca^++^ currents and presynaptic proteins. This increases or suppresses, respectively, the release of the neurotransmitter glutamate from the synaptic body to activate the afferent acoustic axons (cartoon of Figure 4 and original micrographs of Figure 8 and Figure 9). 

All these mechanisms are connected in a chain of time-locked events within OHC and provide the link between specific sound frequencies and specific amplification of mechanical events within the Corti’s organ. These mechanical events produced by OHC are seminal in providing the selective amplification and tuning of sounds to reach high tonotopic discrimination by recruiting specific clusters of IHC. In fact, as we shall see, the mechanical activity of OHC (MET) adds to the mechanical effects (radial shearing) produced by sound on the basilar membrane (and mostly the reticular lamina). As a consequence, when sound reaches the cochlea, direct pressure pushes or pulls the basilar membrane (reticular lamina), which is further tilted by the contraction/elongation of OHC. 

## 4. OHC in the Context of the Corti’s Organ

**Figure 10:**
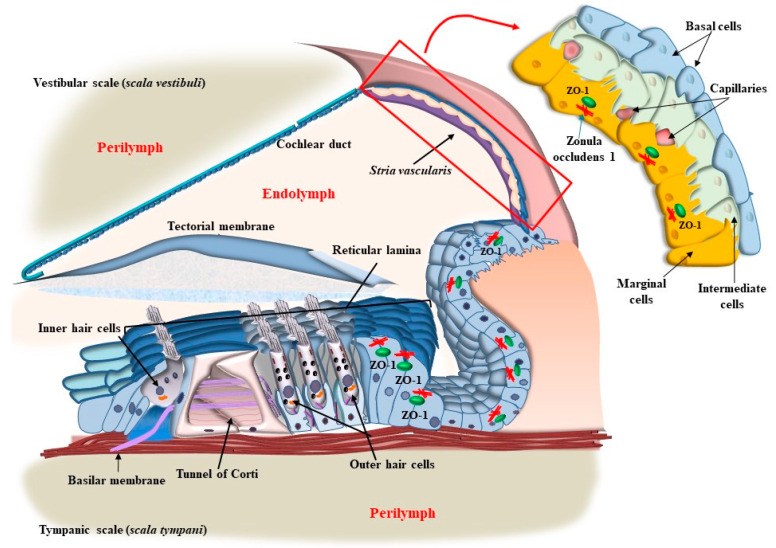
The anatomical and functional coupling between the organs of Corti and *stria terminalis*. The organ of Corti features an RL, where both HC and supporting cells are bound by tight junctions, which typically express the protein Zonula Occludens 1 (ZO1). This allows the diffusion between various cells of the Corti’s organ of electrical activity through the diffusion of various ions. This process is also important to bind the organ of Corti with the vascular epithelium placed on the lateral wall of the cochlear duct, named the *stria vascularis*. In fact, the *stria vascularis* is able to produce endolymph through an active mechanism where ions are concentrated. This process is initiated at the level of the Corti’s organ when, following re-polarization, a transfer of ions from the Corti’s organ through tight junctions occurs. Within the *stria vascularis*, the endolymph content is secreted by the cochlear duct from the superficial cells, which constitute the marginal cells (stained in yellow).

When observing the Corti’s organ (Figure 1 and Figure 10), the BM is placed to limit the tympanic scale. In such a position, the BM directly sustains supporting cells such as Deiter’s, pillar, Hensen’s, and Claudius’ cells, providing an indirect mechanical pivot to sustain HC, which instead is placed on the cell membrane of supporting cells. Differing from the BM, the apical membrane of both HC and supporting cells corresponds to a single level, where each cell is connected to the other through tight junctions to generate a mosaic-like RL. The tight junctions of RL are key to sealing the Corti’s organ and keeping the endolymph as a separate compartment from the perilymph (Figure 10).

**Figure 11:**
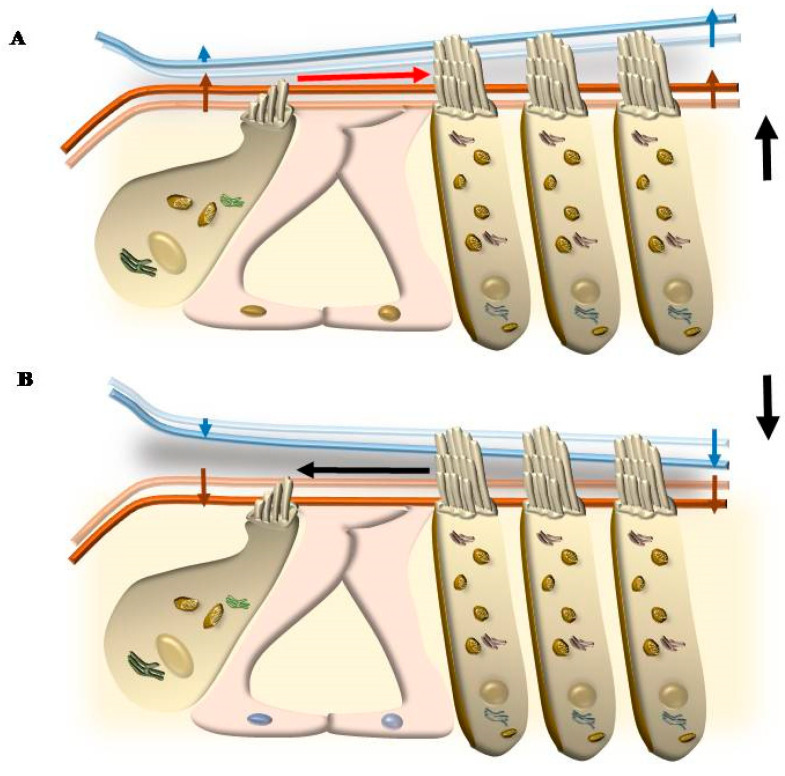
The model of radial shearing. The passive role of RL, which tilts differently from TM, leads to a shearing of endolymph radially in the cochlear duct. In fact, the RL tilts according to a wider amplitude compared with the rigid TM. Such different tilting is also induced by different pivots where RL and TM are attached. In fact, while the RL pivots to inner pillar cells, the TM pivots to the spiral limbus. This generates variations in the space between RL and TM depending on the direction of the acoustic wave. In fact, when pressure tilts up the RL and TM from the *scala tympani* (**A**), the wider tilting of the RL compared with the TM generates a wider space, which increases radially from the modiolus to the *stria vascularis*. This progressive opening of the space between TM and RL during the upward pressure from the scala tympani squeezes the endolymph from the modiolus to the *stria vascularis*. This drive produces the shearing of SC of both IHC and OHC from the shortest to the longest, which in turn generates an excitation of both types of HC. On the contrary, when sound pressure propagates within *scala vestibuli* (**B**), the downward pressure over TM and RL generates a drive of endolymph in the opposite direction, which produces hyperpolarization of HC.

Thus, two membranes, the BM and RL, are placed at the two borders of the Corti’s organ, and they tilt differently during acoustic stimulation. The BM is placed at the border between the tympanic scale and the Corti’s organ. Over the BM, the supporting cells, including the pillar cells and Deiter’s cells, are inserted (Figure 10). The RL is placed at the apical surface of both supporting cells and the HC, where SCs are inserted. At this level, gap junctions are mainly composed of the protein Zonula Occludens 1 (ZO-1) ceil endolymph from the perilymph, which is present between HC and supporting cells (within Nuel’s spaces) and within the tunnel of Corti (Figure 10). During the propagation of sound waves, the RL tilts much more than the BM. Thus, SC placed at the apex of HC represents the key structures sensing sound. In fact, differing from other domains of HC, the apical membrane gifted with SC is surrounded by endolymph and anchored to the base of inner pillar cells, while the TM is pivoting at the level of the spiral limbus and hanging over the Corti’s organ (Figure 1 and Figure 10). According to the classic model of radial shearing, due to the different pivot and tilting of RL compared with TM, when an upward movement of the BM and RL is produced by sound (towards the TM), a depolarization of HC occurs, while a downward movement of the RL (away from the TM) produces HC hyperpolarization (Figure 11).

This phenomenon is based on the tilting of the BM and, mostly, the RL [15]. In fact, recent studies carried out using low coherence interferometry indicate that tilting of the RL [16] is the key phenomenon compared with tilting of the BM [17], which was previously considered fundamental. In particular, evidence is provided that the RL undergoes wider movements compared with BM [18,19], which makes mechanical distortions of the RL more relevant compared with distortions generated within BM. The wider tilting of the RL makes it sensitive to a wider tuning following the acoustic stimulus, and it extends several octaves below the site-specific tonotopic area [20].

Indeed, these recent findings lend substance to previous data showing that motions of the BM are not essential to improving threshold sensitivity to sounds. In fact, when impeding the motion of the BM, leaving intact the tilting of RL, no change in the threshold of sound sensitivity was measured by Braun (1996) [21]. This is seminal to understanding why the neuropathology of tinnitus involves OHC SC as part of the RL without any noticeable alteration of the BM.

In detail, the early cytopathology of tinnitus involves the apical domain of OHC, mostly SC, which anchors to TM. Thus, it is not surprising that inappropriate coupling between RL and TM is the key alteration that leads to tinnitus. 

## 5. Mechanisms That Produce the Movement of SC

### 5.1. Radial Shearing

In line with this, the classic view about IHC and OHC stimulation is based on the anchorage of OHC to TM, which is expected to keep the gap between TM and RL constant during the tilting of the Corti’s organ induced by sounds. The different pivot for tilting of RL (the inner pillar cells) compared with the pivot of TM (its insertion in the spiral limbus, Figure 1) generates a gap during the tilting of both membranes, which is considered the main drive for endolymph to move SC of both OHC and IHC during radial shearing. Thus, the different pivots of TM and RL determine the shearing of endolymph during the tilting of these membranes. Since the tilting of the RL is in excess compared with the tilting of the TM, when the sound passes the tympanic scale, the RL and TM move up to a different extent. In fact, the upward tilting of the RL exceeds the upward tilting of the TM. This produces a narrowing of the RL-TM gap, which is more pronounced where HC does not anchor TM (towards the modiolus) over IHC. In contrast, the anchorage of OHC SC to TM pushes a wider tilting of TM over OHC. The overall effect is a compression of the space over IHC compared with the space over OHC (the IHC RL-TM gap compared with the OHC RL-TM gap). This generates a gradient of endolymph, which shears the SC of both IHC and OHC radially from the modiolus towards the stria marginalis, thereby producing HC excitation (Figure 11). When the compressive phase of sound passes the vestibular scale, the opposite phenomenon takes place (Figure 11). In fact, the downward tilting of the TM over the area of OHC is more pronounced compared with the downward tilting of the TM over the area of IHC, which opens the RL-TM gap medially over IHC and leads a radial endolymph dragging towards the modiolus, which inhibits both OHC and IHC (see cartoons in Figure 11). 

To synthesize the concept of radial shearing, the close relationship between TM and OHC and the peripheral placement of OHC compared with IHC enable wider upward and downward movements of TM over OHC compared with TM over IHC.

### 5.2. OHC Motility (Length Modulation) Adds on Radial Shearing

The concomitant contraction of OHC has modified such a simple view. In fact, according to this simple view, the endolymph flow would solely depend on a radial shearing along the RL due to different pivots and wider tilting of the RL compared with TM. In this way, the radial shearing would act mechanically to deflect OHC stereocilia according to RL movements, which in turn generate a dragging of viscous endolymph to deflect free-floating stereocilia of IHC. 

**Figure 12:**
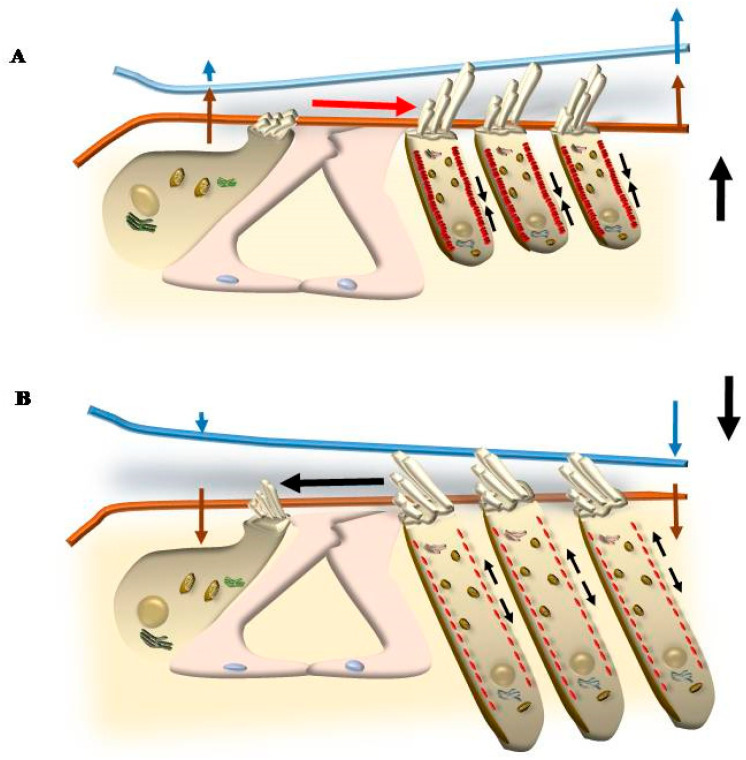
The role of OHC contraction in driving endolymph. During OHC contraction, the gap between TM and RL is increased over OHC (**A**), which drags more endolymph to move from the smaller volume above IHC towards the greater volume above OHC. This leads to an excitatory deflection of the IHC stereocilia. On the contrary, during OHC elongation (**B**), the space between RL and TM over OHC is compressed, and the endolymph flow is directed towards IHC, which deflects in the inhibitory way IHC stereocilia. This is the active role of OHC in squeezing the endolymph and enhancing radial shearing. Such a squeezing also occurs in the perilymph of Corti’s tunnel, which squeezes the pressure orthogonally towards the apex of the cochlea (see text).

Such a model is now implemented by novel data, which indicate that the role of OHC is not limited to amplifying the tilting of RL generated by the propagation of sound. In fact, contraction and elongation of OHC modify the space between RL and TM (the RL-TM gap), which is not as steadily locked as previously thought. As a matter of fact, when OHC modifies their length, the gap between TM and RL is modified as well. In detail, as shown in Figure 12, during OHC contraction, the gap between TM and RL is increased over OHC, which drags more endolymph to move from the smaller volume above IHC towards the greater volume above OHC. This leads to an excitatory deflection of IHC stereocilia. On the contrary, during OHC elongation, the space between RL and TM over OHC is compressed, and the endolymph flow is directed towards IHC, which deflects IHC stereocilia in an inhibitory way (Figure 12). 

The effects of OHC contraction enhance the radial drive of endolymph, thus amplifying the deflection of IHC SC. 

A further consequence, which is produced by the active contraction of OHC, is the rotation of the RL around its pivot, the inner pillar cells. This generates a rotative torsion (clockwise or counterclockwise) of the RL, both perpendicular to radial shearing and still occurring on the plane of the RL. This further squeezes the endolymph, adding another drive to move the cilia of the IHC in the excitatory/inhibitory direction [11].

OHC motility may also spread the endolymph flow in the longitudinal direction (perpendicular both to radial shearing and RL rotation). In fact, contraction of OHC also reduces the space between RL and the BM, which compresses the thickness of the Corti’s organ, which in turn reduces locally the volume of the Corti’s tunnel. This reduces the spaces between HC and supporting cells, including pillar cells. This generates a longitudinal drive along the tunnel of Corti and space of Nuel in a direction that is opposite to the perilymph drive (towards the apex when the sound passes the tympanic scale towards the basal turn). This longitudinal squeezing adds to radial shearing, OHC contraction, and RL rotation in producing the complex mechanisms leading to IHC stimulation [11]. 

**Figure 13:**
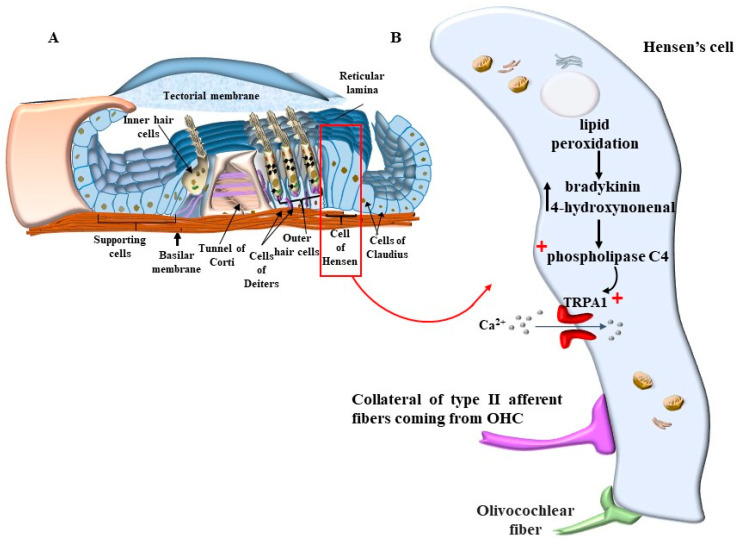
The additional role of Hensen’s cells in HC excitation in specific conditions. (**A**) The excitation of OHC is further modified during specific conditions by the activity of Hensen’s cells, which contract according to a specific pattern [22,23,24]. The stimuli acting on Hensen’s cells are both coming from endolymph and efferent innervation. (**B**) Concerning chemical signaling from the endolymph, Hensen’s cells possess Transient Receptor Potential Cation Channel A1 (TRPA1 channels), which are also present on nociceptive nerve endings in the whole body. TRPA1 channel activation depends on the modulation of phospholipase C4, which is strongly affected by ATP, as well as specific chemical species produced during oxidative stress and/or inflammation. Thus, during inflammation or specific cell damage, by-products produced locally or dispersed in the surrounding endolymph may act on phospholipase C4 to activate TRPA1 channels. Among cell stressors sensed by TRPA1 channels, bradykinin or endogenous products of lipid peroxidation such as 4-hydroxynonenal (4-HNE) are relevant triggers. This makes the activity of Hensen’s cells partly dependent on the amount of cell damage. Activation of TRPA1 channels in Hensen’s cells triggers persistent Ca^++^ responses, which spread through gap junctions to neighboring cells, including HC. Nonetheless, activation of Hensen’s cells may also happen independently of TRPA1 channels through the innervation of specific effent fibers. In fact, the efferent innervation of Hensen’s cells is produced partly by olivo-cochlear fibers, and a considerable amount of nerve endings to Hensen’s cells come from the collateral of type II afferent fibers synapsing OHC. In this way, pathological events affecting OHC are likely to alter the activity of Hensen’s cells, thereby amplifying cochlear degeneration.

OHC motility alters this process, also considering the contraction and elongation of the sole stereocilia. In fact, contraction and elongation of stereocilia, apart from the whole OHC, may modify the strength of anchoring RL to TM, which is more effective during contraction and looser during elongation. Such a variation in anchoring OHC to TM affects the radial shearing. These effects are further modified considering the contraction of Hensen’s cells, which occurs according to a pattern that is different from OHC [22,23,24]. In fact, Hensen’s cells contract according to different stimuli (Figure 13). 

In detail, as recently reported, Hensen’s cells possess Transient Receptor Potential Cation Channel A1 (TRPA1 channels, similar to those of nociceptive neurons). Activation of TRPA1 channels in Hensen’s cells triggers persistent Ca^2+^ responses, which spread through gap junctions to neighboring cells, such as pillar and Deiters’ cells [25]. In this way, long-lasting Ca^2+^-dependent contractions of a number of supporting cells are produced. It is essential to consider that, in baseline conditions, the role of Hensen’s cells is not relevant. In fact, TRPA1 channel activation depends on the modulation of phospholipase C4, which is strongly affected by ATP, as well as specific chemical species produced during oxidative stress and/or inflammation. Thus, during inflammation or specific cell damage, by-products may act at phospholipase C4 to activate TRPA1 channels. It is frequent that among cell stressors sensed by TRPA1 channels, bradykinin or endogenous products of lipid peroxidation such as 4-hydroxynonenal (4-HNE) are relevant triggers. This makes the activity of Hensen’s cells partly dependent on the amount of cell damage (Figure 13). 

**Figure 14:**
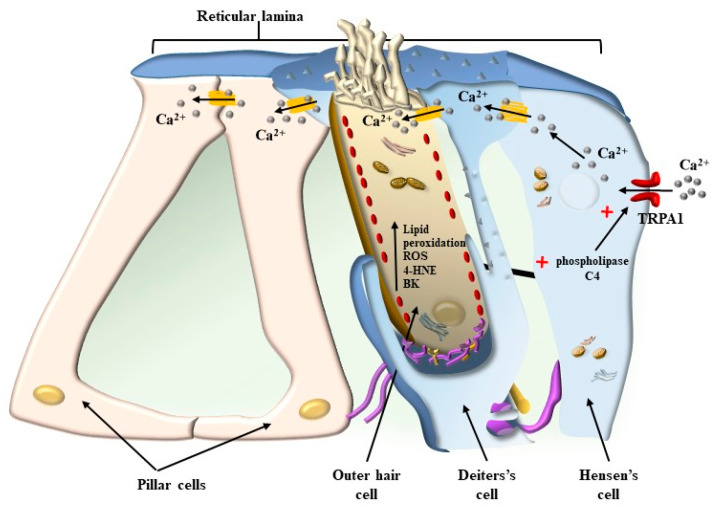
Spreading of excitation from Hensen’s cells to the external layer of OHC. There is a strict relationship between Hensen’s cells and the external layer of OHC. In this way, excitation occurring within Hensen’s cells may directly propagate through a tight junction to OHC.

This may also be relevant in physiological conditions, mostly upon over-activation of the inner ear, when high oxygen consumption takes place in the presence of loud noise. This is exacerbated in the course of metabolic disorders where lipid metabolism and mitochondrial turnover are impaired. The recruitment of TRPA1 receptors in Hensen’s cells does not appear to be relevant for physiological hearing since knockouts for TRPA1 possess a higher threshold for peripheral pain but no change in hearing threshold [26]. It is more likely that the recruitment of TRPA1 receptors may become essential in critical conditions during a number of pathological processes of the inner ear. The recruitment of Hensen’s cells may also be triggered by efferent innervation. In fact, Hensen’s cells possess dense efferent fibers [26] (Figure 13). This may explain how abnormal activity of Hensen’s cells may be induced by stressful events, which may occur at the onset of tinnitus, or how the occurrence of tinnitus may represent a secondary effect in the course of central neurodegeneration. In fact, the innervation of Hensen’s cells, along with Deiter’s cells, is produced partly by olivo-cochlear fibers [27]. However, a considerable amount of nerve endings in Hensen’s cells derive from the collateral of type II afferent fibers coming from OHC. In this way, pathological events affecting OHC are likely to alter the activity of Hensen’s and Deiter’s cells, thereby amplifying cochlear degeneration. As shown in Figure 14, there is a close relationship between Hensen’s cells and the external layer of OHC. In this way, excitation occurring within Hensen’s cells may directly propagate through a tight junction to OHC. This is particularly relevant for the external layer of OHC, which is more strictly coupled with Hensen’s cell activity. 

The ability of supporting cells to participate in the motion of the Corti’s organ indicates how intricate the outcome of a sound stimulation is on the activity of the auditory receptors of the inner ear. In fact, the motility of OHC is believed to amplify the motions of IHC, thereby increasing hearing sensitivity. Nonetheless, the overall motion of Corti’s organ, which takes place approximately 2–6 octaves below, is complex since the motion produced by OHC, Deiter’s cell, and Hensen’s cell is much larger than the TM and RL [28]. This further complicates the drives acting on the IHC SC. One should consider that the contribution of Hensen’s cells, despite not being essential in baseline conditions, may be significant when high amounts of detrimental by-products of oxidative metabolism are produced. In fact, TRPA1 receptors, as widely described for Toll-like receptors, are sensitive to a number of chemical species that are generated by the metabolic distress of the Corti’s organ. Thus, the drive of SC movements generated by Hensen’s cells should be specially dissected in the course of pathological phenomena affecting the Corti’s organ during OHC degeneration.

### 5.3. Further Drives to Move IHC SC

**Figure 15:**
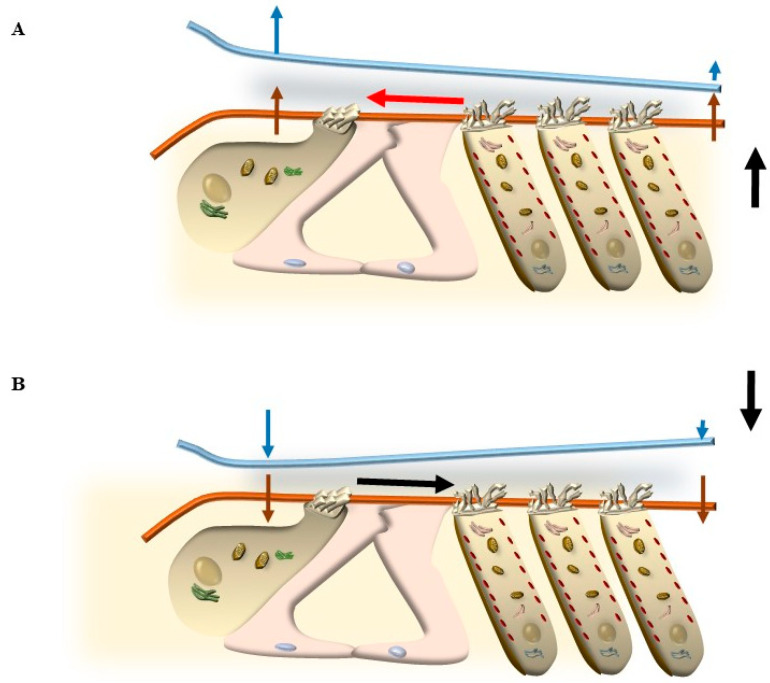
Pathological reversal of radial shearing. When considering early SC alterations, which may induce tinnitus, some structural changes are common. These changes may involve a number of molecules and organelles of HC, although the key effect consists of the loss of rigidity, which alters the endolymph drive (stereocilia slant drive), or a paradoxical increase in rigidity in the attachment of SC to the TM, which alters again the endolymph drive (TM push/pull drive) [11]. In these conditions, the abnormal role of OHC is also effective in the opposite direction. When a stimulus acts upward from the scala tympani, a latero-medial deflection, which leads to excitation in normal conditions, shifts to produce a paradoxical inhibition (**A**). On the contrary, when the stimulus occurs downward of the RL from scala vestibuli, the OHC does not elongate and does not pull down the TM. Still, considering that the tilting of the RL surpasses the slight deflection of the TM, a wider RL-TM gap occurs over the OHC, which drags endolymph drive externally from the modiolus to exert a paradoxical stimulation of the IHC (TM pull drive) (**B**). This may be responsible for a reversal of IHC stimulation and powerful activity in the acoustic nerve off-phase of acoustic stimulation. In the course of peripheral tinnitus and presbyacusis, such a paradoxical phase reversal (TM push and TM pull drives) is expected to play a role in generating cyclic phantom noise and hypoacusia, just like it occurs during peripheral tinnitus in the course of OHC degeneration. In fact, the reversal of IHC activation explains the generation of noise perception in the absence of stimuli and, conversely, a decreased perception of acoustic stimulation when stimuli do occur.

Among the various endolymph drives being summarized here, squeezing of endolymph due to contraction of OHC in the presence of an upward deflection of RL is expected to be the predominant drive along with classic radial shearing in producing the activation of IHC, which determines the acoustic stimulation in baseline conditions. This explains why, in the presence of damage to OHC SC, the direction of endolymph dragging promoted by sound waves may be pathologically reverted, as shown in Figure 15.

Even posing the RL as the main vibrating structure of the Corti’s organ, the nature of the finest mechanisms that lead to the stimulation of IHC following amplification operated by OHC remains a matter of debate. Novel findings indicate that such a deflection may also depend on the relationship between TM and IHC SC [29]. Thus, in addition to anchoring OHC SC to TM, IHC SC appears to have some important connections to TM. This is not merely a mechanical phenomenon since TM works as a store for Ca^++^ needed by both IHC and OHC. In fact, in their elegant manuscript, Hakizimana and Fridberger (2021) [29] provide evidence that Ca^++^-rich filamentous structures, named “Ca^++^ ducts,” connect the TM to IHC stereocilia to enable direct stimulation of IHC by the TM. In this study, the binding of IHC to TM is further documented to involve electrical effects. In fact, TM possesses the highest Ca^++^ concentration measured in the whole cochlea, which enables a direct Ca^++^ influx from TM to the apical membrane of both IHC and OHC. This enables the entry of high Ca^++^ levels from TM towards both OHC and IHC through calcium ducts, which are independent of the chemical composition of the endolymph [29]. This is key when considering that, upon stimulation, the request of Ca^++^ to feed the hearing process ranges around a concentration of 100 μM [30], which is way in excess compared with the Ca^++^ concentrations, which are measured in the endolymph (20 μM) [31]. The ability to force and tunnel Ca^++^ entry through Ca^++^ ducts from TM to OHC and IHC would solve this enigma. The relevance of TM as the main Ca^++^ store in the cochlea is in line with the later onset of TM during phylogenesis as a mechanism developed to further strengthen the effects of endolymph on both OHC SC and IHC SC [32]. The protein structure of Ca^++^ ducts remains largely non-explored for OHC and mostly ignored for IHC. Nonetheless, Ca^++^ itself is essential to anchoring TM to SC, as shown by findings that, upon Ca^++^ removal, the linking between TM and RL is greatly reduced. One protein known to take part in this process is sterocilin, which anchors TM to OHC [30], while a similar role was hypothesized for otoancorin to IHC [33]. This explains why specific defects in the protein composition of TM during metabolic dysfunction affecting protein turnover and protein clearance may lead to tinnitus and pesbyacusis.

The presence of an intimate electro-chemical connection, in addition to mechanical interaction between TM and IHC, adds to a variety of mechanisms that are likely to play a role in transferring the activity of OHC into the generation of IHC excitation, which in turn produces action potentials traveling along the acoustic nerve.

The plethora of factors that influence the activity of the acoustic nerve makes it intricate to dissect the molecular mechanisms that operate in the course of tinnitus and whether a final common pathway may be responsible for this symptom. Still, in considering the fine neuropathology of tinnitus and presbyacusia, some data are predominant, which indicate an early impairment of OHC SC (which also implicates a defective anchoring of SC to TM) in all kinds of persistent tinnitus.

## 6. The Main Source of Tinnitus

Most cases of tinnitus are likely to be generated by inappropriate mechano-electrical activity/transduction (MET), which is triggered at the tip and the side domains of OHC SC [8,34]. Such impairment can be associated with a number of causes, such as an abnormal amount of oxidative species and/or elevated glutamate levels at the afferent synapse of outer hair cells or Hensen’s cells. These phenomena, in turn, are concomitant with aging and hearing loss [8], and they are exacerbated by environmental distress such as loud noise, neck injury, trauma, and ototoxicity [35,36]. Despite the list of detrimental factors associated with tinnitus being rather general and simplistic, the neuropathology, in most cases, reflects overlapping morphological alterations. This consists of the engagement of the apical and lateral domains of the OHC of the Corti’s organ, where SC is attached. These findings call for a specific focus on this structure. In fact, in order to comprehend the genesis of tinnitus and its relationship with hearing loss, constant damage to the HC needs to be properly deciphered. Among auditory cells, HC is mostly affected, and within the group of HC, OHC is much more sensitive than IHC to tinnitus-related degeneration. This is key since, unlike other cell types of the inner ear, HC in mammalians cannot be substantially replaced [37], which leads to persistent defects of auditory function. This is why, in most cases of degenerative tinnitus and presbyacusis, there is a chronic reiteration of detrimental stimuli, and the onset of tinnitus occurs later in life. The earliest damage that involves OHC is placed at the level of OHC SC. In several cases, SC is lost, although such a loss is anticipated by impaired function of SC, which can be interpreted based on the mechanisms of auditory stimulation that were reported in the first part of the present manuscript.

When considering early SC OHC alterations, which may induce tinnitus, some structural changes are common. These changes may involve a number of molecules and organelles of HC, although the key effect consists of the loss of rigidity that alters the endolymph drive (stereocilia slant drive) or a paradoxical increase in rigidity in the attachment of SC to the TM that alters the endolymph drive (TM push/pull drive) [11].

### 6.1. The TM Push/Pull Drive in the Course of Degeneration

When damage to OHC SC occurs, it is expected that an upward movement of the RL does not stimulate the tip-link to produce a contraction of OHC, which may fail to produce an increase in the RL-TM gap over OHC. In this way, since the TM is less elastic than the RL, a compression occurs over OHC, which squeezes endolymph towards the modiolus, thus inhibiting IHC instead of producing an excitation (Figure 15). In these conditions, the abnormal role of OHC is also effective in the opposite direction. When a stimulus acts downward on the RL from scala vestibuli, the OHC SC does not elongate and does not pull down the TM. Still, considering that the tilting of the RL surpasses the slight deflection of the TM, a wider RL-TM gap occurs over the OHC, which drags an endolymph current externally from the modiolus to paradoxically stimulate the IHC (TM pull drive) [11]. This may be responsible for a reversal of IHC stimulation and powerful activity in the acoustic nerve off-phase of acoustic stimulation. In the course of peripheral tinnitus and presbyacusis, such a paradoxical phase reversal (TM push and TM pull drives) is expected to play a role in generating phantom noise and hypoacusia, just like it occurs during degenerative tinnitus. In fact, the reversal of IHC activation explains the generation of noise perception in the absence of stimuli and, conversely, a decreased perception of acoustic stimulation when stimuli do occur. Another mechanism that may lead to a reversal of the physiological auditory response is the stereocilia slant, which often occurs in genetic disorders affecting the protein composition of stereocilia. Similarly, in the course of degenerative phenomena, the occurrence of altered protein turnover leads to a misfolding of key proteins of SC, which may also result in the stereocilia slant.

### 6.2. The Stereocilia Slant Drive in the Course of Neurodegeneration

This occurs when the angle between the SC and the cuticular membrane of OHC in a resting state is greatly reduced compared with the classic 90°. Such a condition typically occurs during the early stages of cochlear degeneration, when stereocilia lose their stiffness, leading to tinnitus and hypoacusia (Figure 6). The slant drive may appear when selective damage of OHC-SC is evident as an abnormal slanting of SC or increased deflection of SC due to a loss of SC rigidity produced by disarrangement of key structural elements. These phenomena are common early steps during the degeneration of OHC. In both cases, the effects of OHC contraction are lost, and OHC plays a passive role [11]. This greatly reduces/altes/reverses the role of OHC as an amplifier. As a consequence, upward deflection of BM may squeeze endolymph towards the modiolus, leading to inhibition of IHC. On the other hand, downward deflection is expected to produce an excitation of IHC (Figure 15). Both conditions represent a pathological reversal and suppression of physiological mechanisms of stimulation and may lead to tinnitus and hypoacusia.

Thus, the TM push/pull drive, as well as the stereocilia slant drive, may be contingent on specific pathological conditions [11]. In fact, when degeneration of OHC is progressing, starting from SC, it is likely that such an abnormal stimulation occurs quite often, leading to phantom noise and/or an increased hearing threshold. 

## 7. Which Molecular Alterations Early Affect OHC SC to Generate Tinnitus?

A number of detrimental factors may produce tinnitus, and a trivial list does not help very much to understand whether intimate alterations occur to produce the symptom or whether a final common pathway exists among the plethora of contributing factors. Most factors inducing persistent tinnitus as a primary effect produce stimulation of cochlear HC. In fact, from a general perspective, an excess of such stimulation (loud noise exposure) may be responsible for cell dysfunction and cell degeneration. Thus, repeated exposure to an excess of acoustic stimulation occurring during the course of repeated acoustic trauma or chemicals, including ototoxic drugs, may converge in producing primary damage to OHC SC. These patients represent the vast majority of those complaining of tinnitus, and they often feature damage in the inner ear, which consists specifically of the loss of the main activities we previously introduced as typical of OHC. In fact, consistently, with early dysfunction in SC, the loss of MET is the earliest effect. This is due to an impairment of the activity of tip-links, which fail to stretch the membrane of the contiguous cilium. The loss of such a coupling is key in altering the mechanisms that sustain the function of SC, making it more likely the occurrence of abnormal contacts between TM and RL, as we previously described as “The TM push/pull drive” and “The stereocilia slant drive” [11]. In fact, in both cases, the contact between OHC and TM produces abnormal, paradoxical events. In the case of the push/pull drive, the harmonic shearing induced by the TM on RL does not occur, and SC is not sheared during the contact between RL and TM but persists instead in their orthogonal position without deflection, which keeps the RL-TM gap rigid. These abnormalities may include a loss of elastic properties of SC, a tenting of SC, or a dimpling of SC within the TM [11]. During TM dimpling, the upward deflection of the RL leads the stereocilia to dimple an area of the TM. Such a dimple due to an altered structure of TM reduces the baseline gap between the RL and TM, making the indentation and attachment of the cilia to the TM less effective in keeping the RL-TM gap steady during upward and downward movements of the RL. In the case of tenting, the structure of the SC is altered, where the actin component is intact but the core of the SC is not properly anchored to the actin-filled periphery. This generates a lack of effective anchoring between the central and peripheral parts of the cilium, which does not allow the periphery to drag the TM during the movements of the RL. This impairs the downward movements more but has little effect on the upward movements of the RL-TM complex. Damage to OHC stereocilia produces a displacement of the TM, which may produce vigorous abnormal contact with IHC, thus producing baseline inappropriate activity. The loss of OHC can be initiated either by an excess of calcium and/or abnormal protein accumulation. The excess in calcium level is bound to strong reiterated acoustic stimulation, which typically occurs in the genesis of tinnitus and presbyacusis. Indeed, an altered OHC SC structure is expressed by an altered coupling between OHC SC and TM. This is mostly due to altered OHC SC, although a primary alteration in the structure of the TM may greatly contribute to tinnitus.

### 7.1. The Specific Mechanisms Altering OHC SC Structure

#### 7.1.1. Oxidative Stress

As mentioned above, the pathogenesis of tinnitus, or the underlying mechanisms that contribute to its development, is a complex and multifaceted process. Several factors, including aging, acoustic trauma, ototoxic medications, metabolic, otologic, psychiatric, and neurological diseases, as well as oxidative stress, can contribute to the onset and progression of tinnitus [38,39]. The overactivity of the inner ear, as well as metabolic alterations involving mitochondria, are supposed to increase the amount of oxidative species. In these conditions, a number of by-products are generated that are able to activate the TRPA1 channels placed on supporting cells (mostly Hensen’s cells) and auditory cells (Figure 13 and Figure 14). Hensen’s cells are mostly prone to the effects of oxidative species on TRPA1 channels, and a great number of oxidative species are supposed to alter their activity, which spreads to neighboring Deiter’s cells and OHC. In fact, these latter cell types, which are the most prone to stress, possess the highest level of stress-inducible cyclooxygenase 2 COX-2 isoform [40], which is believed to act as a compensation for intense noise exposure to exert a compensatory effect.

In fact, oxidative stress occurs when there is an imbalance between the production of reactive oxygen species (ROS) and the cells’ ability to counteract these compounds. ROS, such as superoxide radical (O_2_), hydroxyl radical (OH^−^), and hydrogen peroxide (H_2_O_2_), are by-products of normal cellular metabolism and are essential for different biological activities. When high ROS levels are produced, pathological alterations occur due to the oxidation of lipids, proteins, and nucleic acids, ultimately inducing cell death [41]. 

The antioxidant defense system, including both enzymatic and non-enzymatic molecules, plays a crucial role in counteracting ROS. The enzymatic system includes, among others, superoxide dismutase (SOD), catalase, glutathione peroxidase (GPx), glutathione S-transferase (GST), glutathione reductase (GR), and thioredoxin (TRX). The non-enzymatic system includes several molecules such as vitamin C, uric acid, bilirubin, and thiol systems, including thioredoxin (TRX) and reduced glutathione (GSH), and organic molecules that contain a sulfhydryl (-SH) group [42].

The inner ear is particularly susceptible to oxidative stress due to its high metabolic activity and limited antioxidant defense [43]. Redox imbalance in the inner ear impacts mostly HC (both IHCs and OHCs), which, upon injury, cannot regenerate [44]. Exposure to loud noise or ototoxic drugs that induce oxidative stress has been shown to result in increased levels of ROS-induced damage to HC and the development of hearing loss and tinnitus.

Thus, an excess of free radicals, ROS, and a lack of antioxidants can be involved in tinnitus. Although it is very indirect and remote evidence, high levels of oxidative species are measured within venous blood drainage from the inner ear in patients with idiopathic tinnitus [45,46]. These include plasma malondialdehyde (MDA) and 4-hydroxynonenal (4-HNE) as markers of lipoperoxidation, myeloperoxidase (MPO) as a marker of protein damage, and glutathione peroxidase (GPx) as an enzymatic antioxidant. High aldehyde levels, such as MDA and 4-HNE, were measured in the jugular blood of patients suffering from tinnitus compared with controls. It is remarkable that the very same aldehydes are powerful stimulators of the recently described TRPA1 channels occurring on the plasma membrane of Hensen’s cells (Figure 13 and Figure 14). In fact, as reported above, Hensen’s cells may produce stimulation of OHC [22,23,24], which depends on the activation of the TRPA1 channel, which triggers persistent Ca^++^ responses, spreading to other cell types in the Corti’s organ [25] to generate long-lasting Ca^++^-dependent contractions of a number of cell types. It is essential to consider that TRPA1 channel activation depends on the modulation of phospholipase C4, which is strongly affected by ATP as well as specific chemical species produced during oxidative stress. Thus, specific compounds generated by lipid peroxidation, such as 4-hydroxynonenal (4-HNE) [47,48], may act on phospholipase C4 to activate TRPA1 channels. This makes the activity of Hensen’s cells key in sensing and mediating the effects of ROS [49]. This is relevant in the course of metabolic disorders where lipid metabolism and mitochondrial turnover are impaired. The recruitment of TRPA1 receptors in Hensen’s cells does not produce hearing loss. However, the recruitment of TRPA1 receptors is essential in critical conditions of the inner ear. The recruitment of Hensen’s cells may also be triggered by a specific innervation. In fact, when a strong activation of OHC is produced during exposure to loud noise, afferent fibers coming from the OHC branch over Hensen’s cells produce their activation [26]. This explains the recruitment of Hensen’s cells in the course of stressful events corresponding to those that induce tinnitus. Again, Hensen’s cells may receive a straight efferent innervation from olivo-cochlear fibers [27], which may correlate the abnormal activity of the brainstem reticular formation, such as that occurring during depression or anxiety, to the onset of peripheral tinnitus. The role of altered motility promoted by Hensen’s cells is likely to be relevant due to the coupling of Hensen’s cells and the outer row of OHC [28]. These latter effects induced by ROS in the endolymph of the inner ear during tinnitus are likely to be relevant to explain discrete and specific acoustic (and vestibular) symptoms since specific molecular events are identified rather than the old concept about a non-defined general effect of ROS within the brain endothelium as being responsible for these specific and focal symptoms.

The cell-specific effects of ROS and the specificity of activators of TPAM1 may explain the relevance of data that correlate oxidative stress with the presence of tinnitus, as documented by the levels of total antioxidant status (TAS), thiols (antioxidant molecules that react with free radicals to prevent oxidative damage), and paraoxonase-1 (PON) activity (an enzyme preventing lipid peroxidation) [38,50,51].

**Figure 16:**
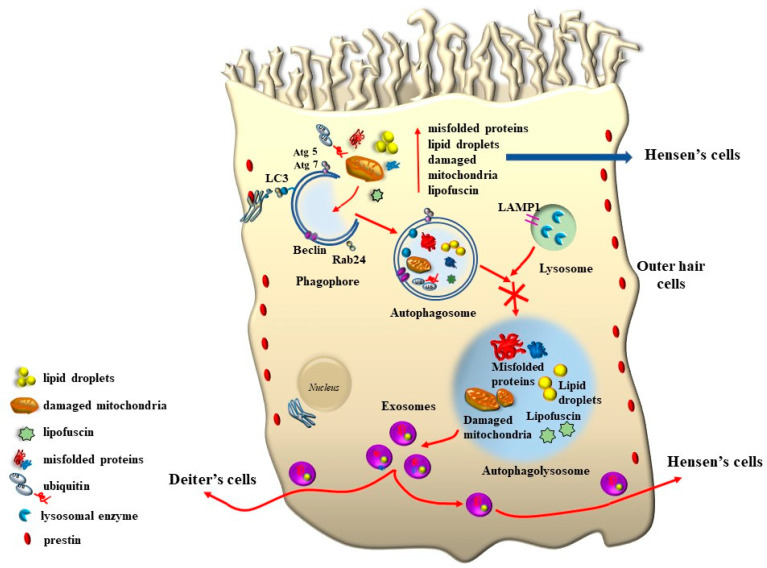
Sub-cellular pathology of OHC during degenerative tinnitus. In the course of degenerative tinnitus, cytopathology mostly affects OHC, where a marked alteration is observed at early stages within SC. These cells are filled with misfolded proteins, lipid droplets, damaged mitochondria, and autophagy vacuoles. These components may also spread nearby to affect Hensen’s cells by acting on the modulation of phospholipase C4 and modifying cell activity. The spreading of these organelles and chemical species may also occur toward neighboring supporting cells through the release of exosomes. Similarly, the endolymph of the cochlear duct may diffuse these components.

The relevance of oxidative species and the need for high energy stores are well connected with the intense activity due to the high-frequency contraction of OHC, which surpasses the large baseline activity of eukaryotic cells (Figure 16). 

Therefore, in connection with the marked oxidative metabolism within OHC, the mitochondrial status is relevant in preserving the integrity of OHC. One may expect that the overproduction of ROS is concomitant with a defect in mitochondrial activity within OHC, and both converge early in the onset of peripheral tinnitus. Moreover, since ROS alters mitochondria and, in turn, damaged mitochondria produce an excess of ROS, a vicious circle may occur. In fact, it is likely that a mitochondrial dysfunction occurring within OHC may be both the consequence and the cause of a high amount of ROS.

#### 7.1.2. Mitochondrial Dysfunction

Mitochondria are the major source of ROS, and mitochondrial dysfunctions in the cochlea are associated with age-related hearing loss and tinnitus [52].

The clearance of altered mitochondria is an important feature that sustains cell survival, which occurs through a specific autophagy process called mitophagy. 

Mitophagy is an important quality control system that selectively degrades damaged mitochondria and helps damaged cells survive under several stressors, including the use of ototoxic drugs. 

In mammals, mitophagy is supported by two key autophagy enzymes: PTEN-induced kinase 1 (PINK1), a serine/threonine kinase, and Parkin (PRKN), an E3 ubiquitin ligase, which works in coordination to target altered mitochondria to be addressed to lysosomal degradation [53]. 

In healthy mitochondria, PINK1 is synthesized in the cell cytosol as a precursor of about 63–68 kDa. It is imported into the mitochondria, where it is cleaved by Presenilin-associated rhomboid-like protease (PARL) to produce a mature form of PINK1 (52–55 kDa). This form is quickly removed from the mitochondria and re-translocated into the cytosol [53]. 

In damaged mitochondria, there is a loss of membrane potential, which leads to depolarization of the inner mitochondrial membrane (IMM). As a result, PINK1 accumulates on the outer mitochondrial membrane (OMM). In this location, PINK1 recruits parkin, activating its latent activity to ubiquitinate and degrade OMM proteins, which leads to the dismantling of altered mitochondria. This triggers the mitophagy process [54]. 

**Figure 17:**
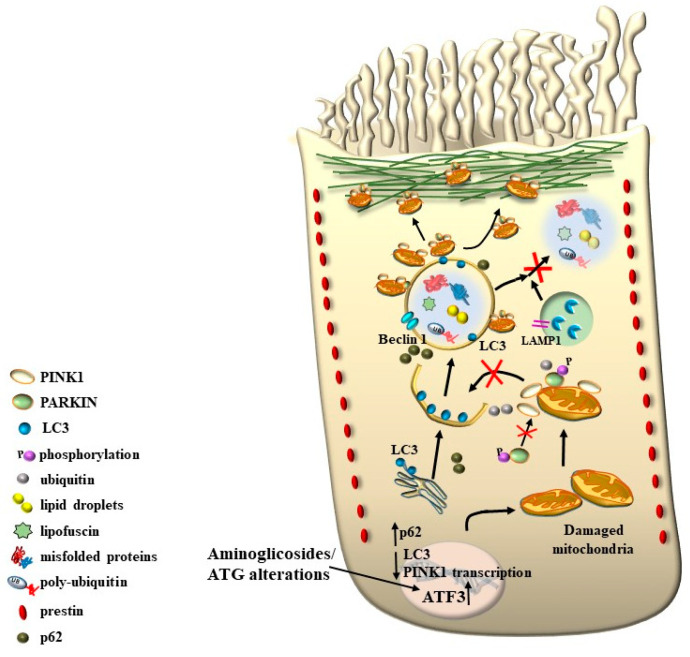
Convergence of various factors in altering the pathology of OHC during tinnitus. Independently of the main causative factor that triggers the onset of chronic tinnitus, the autophagy machinery and the mitochondrial status are consistently altered. Mitochondrial pathology mainly consists of the accumulation of deranged mitochondria, both at the apical and nuclear levels. These findings are concomitant following chronic exposure to ototoxic drugs such as aminoglycosides (AGs) and following repeated acoustic trauma. Nonetheless, specific genetic alterations leading to tinnitus produce the very same findings, which are typically described in the course of age-related degenerative tinnitus associated with presbyacusis.

As expected, PINK1 is expressed at high levels within HCs and plays a crucial role in ototoxicity. House Ear Institute-Organ of Corti 1 (HEI-OC1) cells exposed to gentamicin show increased ROS production and a rapid, acute decline in PINK1 expression. Moreover, the knockdown of PINK1 leads to a decrease in microtubule-associated protein 1 light chain 3 (LC3B), a protein implicated in mitophagy [55,56] (Figure 17).

The critical role of mitochondria in OHC is confirmed by the deleterious effects of ototoxic drugs on the mitochondrial status of these cells. For instance, the knockdown of mitophagy-regulating proteins leads to an increase in cisplatin-induced mitochondrial dysfunction and cytotoxicity in HEI-OC1 cells. The suppression of mitophagy by 3-methyladenine (3-MA) worsens cisplatin-induced ototoxicity. This condition is reversed by carbonyl cyanide m-chlorophenylhydrazone (CCCP), a mitochondrial membrane depolarizing agent [57]. 

The issue of mitochondrial dysfunction in tinnitus, along with specific damage to proteins involved in the motility of SC of OHC, calls for an in-depth analysis of metabolic dysfunctions in degenerative tinnitus and presbyacusis, which may relate to the sophisticated mechanisms of sound transduction of the inner ear with molecular dysfunctions leading to altered sound perception in the course of inner ear degeneration. A crucial kernel in connection with these phenomena is represented by the dysfunction of cell-clearing pathways. These may encompass the deleterious effects of both an excess of oxidative species and damaged mitochondria. In fact, cell clearance is involved in handling damaged mitochondria, altered protein conformation, and lipid peroxidation, which were described as constant detriments in the course of degenerative tinnitus (Figure 16). Therefore, it is mandatory to analyze the potential role of autophagy in the inner ear as a pathway, which may be critical as the final common pathway to modulate the onset and progression of tinnitus and a target of novel therapies. This requires a dedicated paragraph that includes the specific mechanisms described so far. In addition, the appropriate turnover of proteins regulating the structure of the TM and the cytoskeleton of the SC depends on effective autophagy activity. This poses the rationale to infer that altered autophagy may lead to SC slanting or an excess of stiffness, as well as dimpling or tenting of SC within an altered TM. This would connect the mechanisms operating in the course of auditory stimulation to the presence of effective ongoing autophagy for effective protein and mitochondrial turnover.

## 8. The Role of Autophagy in Maintaining the Structure of OHC SC, TM, and Its Failure in Tinnitus 

Solid evidence reports autophagy alterations in the course of several degenerative disorders within the CNS and sensory organs, such as the retina. However, the role of this pathway in the inner ear remains poorly investigated. At present (mid-August 2023), a PubMed search for autophagy and tinnitus sorts 6 manuscripts, while autophagy in the whole inner ear sorts only 99 manuscripts. In fact, this represents an emerging field in hearing research. The relevance of autophagy within HC directly applies to specific effects within HC of the cochlea and labyrinth. This is mostly important in the process of defining novel research directions and planning future therapeutic approaches for diseases of the inner ear that are still lacking effective therapies. This is the case of tinnitus, either alone or concomitant to vestibular syndromes such as the Meniére syndrome or the plethora of Meniére-like syndromes. 

One key element that makes autophagy so promising in hearing research is the relevant role of mitochondrial turnover, which is driven by a subtype of autophagy defined as mitophagy (Figure 17). 

**Figure 18:**
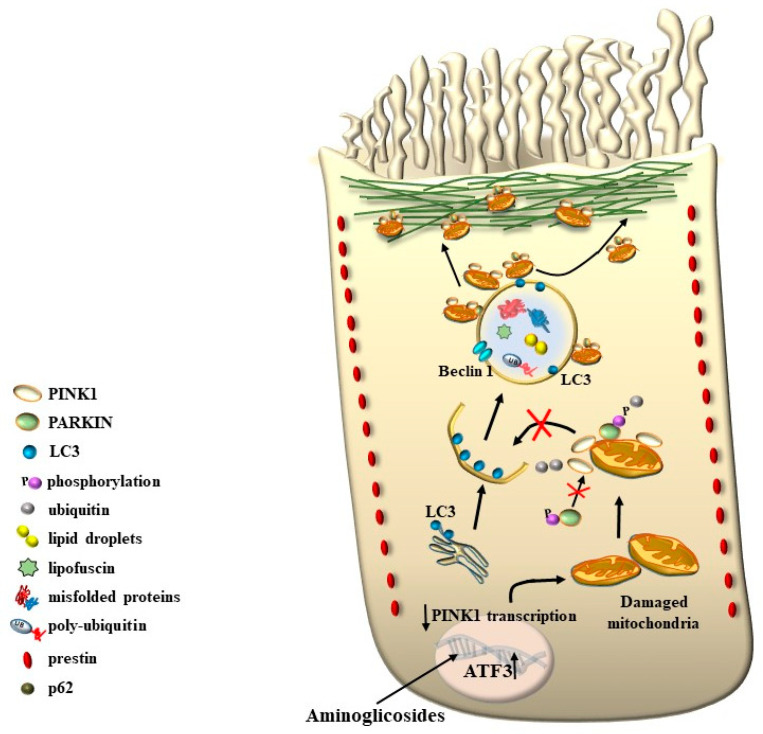
Pathways involved in tinnitus-associated mitochondrial damage. In most cases, including chronic exposure to ototoxic drugs, the marked mitochondrial alteration is characterized by an altered autophagy-dependent Activating Transcription Factor 3 (ATF3) pathway, which in turn disrupts the equilibrium of key proteins involved in mitochondrial turnover such as PINK1 and parkin, along with other molecules that regulate mitochondrial fission and fusion.

Again, the abundance in the cochlea of long-lived proteins (LLP) with either single or multiple lifetimes makes the mechanisms of protein clearance through autophagy very critical to sustaining cochlear integrity. In a manuscript published a few weeks ago, Savas (2023) [58] provides a thoughtful overview of the prominent role of autophagy and ubiquitin-dependent systems, such as the proteasome, in sustaining the integrity of a number of structures in the inner ear. In fact, impaired protein lifespans due to altered protein degradation may be essential in causing acquired degenerative tinnitus and hearing loss. Considering the proteasome and autophagy, the clearance of LLP is mainly operated by autophagy since the proteasome is more involved in degrading short half-life proteins. Therefore, it is not surprising that protein degradation through autophagy is both seminal and articulated in sustaining cochlear function both in baseline and degenerative conditions, leading to tinnitus and deafness. The relevance of autophagy and clearing systems should not be considered a parallel phenomenon aside from ROS and ototoxic compounds in inducing degenerative tinnitus (Figure 18). However, autophagy encompasses a variety of specific mechanisms. In fact, as recently shown by Mu et al. [59], cisplatin administration alters the expression of Forkhead box G1 (*FOXG1*), which is essential to promoting the viability of HC by promoting autophagy. The suppression of autophagy, which is induced by cisplatin, is due to its epigenetic suppression of *FOXG1*. These alterations produce an increase in oxidative species, which exceeds the suppressed buffering effects of autophagy within HC (Figure 17 and Figure 18). This is responsible for the onset of tinnitus and hearing loss. Thus, as hypothesized at the beginning of the manuscript, various potential mechanisms causing tinnitus and presbyacusis may ultimately converge into final common pathways where loud noise, ototoxic drugs, oxidative species, mitochondrial alterations, and aging have synergistic effects. This final pathway consists of impairing fundamental steps in OHC metabolism aimed at sustaining a high rate of oxygen consumption.

As shown by Magarinos et al. [60], a few autophagy genes, such as *Becn1*, *Atg4*, *Atg5*, and *Atg9*, may determine cochlear development and integrity [60]. For instance, the absence of Atg5 produces, by itself, a marked hearing loss [61]. In line with the structural alterations of SC, which are early observed in tinnitus and HC degeneration, the lack of an effective turnover of LLP is supposed to alter the contracting structure of SC and OHC. In fact, a number of misfolded proteins aggregate in these cells in the course of degeneration, which leads to a loss of specific functions in the mechanisms of IHC stimulation. For instance, when subjects are exposed to reiterated loud noise, the engulfment of protein-clearing pathways leads to an increase in ubiquitinated misfolded cochlear proteins. This is witnessed by the engulfment of the cell-clearing system, joining ROS overproduction and mitochondrial pathology (Figure 17 and Figure 18). This explains why degenerative tinnitus is accompanied by protein aggregates [62,63]. In line with this, autophagy inhibitors were shown to alter the structure of OHC SC while producing clusters of lipid droplets along with peroxisome impairment [64].

**Figure 19:**
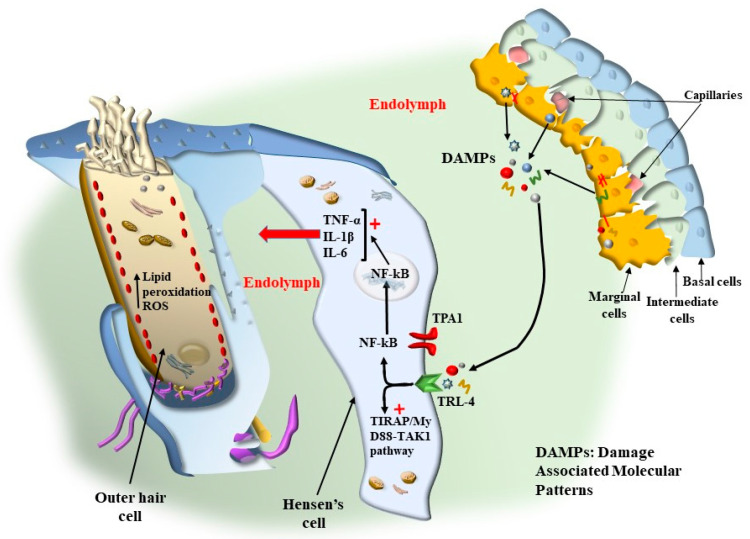
Endolymphatic spreading of deleterious compounds. The presence of exosomes or altered exocytosis may foster the spreading of toxic species between the Corti’s organ and the *stria vascularis*. This may explain the role of endolymphatic alterations in the course of tinnitus, moving the significance of such a phenomenon from mechanical effects toward specific receptor-mediated chemical signaling. In fact, toll-like receptor-4 (TRL-4) on Hensen cells may be activated by damaged associated molecular patterns (DAMPs) produced at the level of the *stria terminalis* or neighboring cells within the Corti’s organ. Similarly, DAMPs may feed back from the Corti’s organ to the *stria vascularis*, thus producing a vicious circle where both specific cytopathology and endolymphatic spreading are connected.

It is remarkable that, mimicking the pathology of tinnitus, which specifically starts with OHC SC, when autophagy is impaired, the earliest alterations occur in the structure of OHC SC. This occurs before any change is observed within IHC. In fact, when producing conditional knockouts for the specific autophagy gene *ATG5* in both IHC and OHC, Fujimoto et al. [61] could demonstrate that mice developed deafness. In this study, although the conditional knockout engaged both IHC and OHC, the damage was much more severe within OHC (just like what happens in the course of degenerative tinnitus). In fact, at post-natal day 14 (P14) of *ATG5* KO, both IHC and OHC develop broken stereocilia (which is supposed to produce stereocilia slunt). At this stage, some frank OHC cell loss is already present in the absence of IHC cell loss (as shown in Figure 5 for the progressive degeneration during tinnitus). When observed at P60, OHCs are almost missing. The specific frailty of OHC compared with IHC to autophagy dysfunction was explored only recently in the manuscript by Zhou et al. [12]. In this work, the authors restricted the conditional knockout to OHC by suppressing the autophagy gene *ATG7*. In these mice, damage to OHC was finely described with early rupture of OHC SC, which was associated with impaired electromotility of OHC. In these conditional *ATG7* knockout mice, synapses were altered early, along with the disorganization of presynaptic ribbons. A dramatic mitochondrial failure was measured, while later on, cell loss and deafness occurred. When analyzed at the subcellular level, degenerating OHC was filled with damaged mitochondria, which could not be cleared (Figure 17 and Figure 18). It is remarkable that the impairment of autophagy induced by the conditional knockout of ATG7 could not be restored by increasing autophagy through Atg7-independent mechanisms. Remarkably, experimental silencing of *ATG7* reproduces all the pathological features that are reported within OHC SC in the course of degenerative tinnitus. It is also remarkable that some autophagy-inducing compounds, such as berberin, have the ability to increase Atg7 levels along with Atg5, making it interesting to test this compound in the effort to antagonize OHC pathology [65] (Figure 17 and Figure 18). When conditional Atg7 deficiency was induced, the autophagy pathway was impaired. This led to an increase in LC3-I, a decrease in the LC3-II/LC3-I ratio, and an increase in p62 (SQSTM1). Remarkably, in *ATG7* conditional KO, as much as in patients affected by degenerative tinnitus, cytosolic inclusions develop, which resemble those occurring in central degenerative disorders. In fact, these inclusions extend from OHC to Corti’s ganglion cells, where p62 (SQSTM1) accumulates to form aggregates within Corti’s ganglion neurons. These p62 neuronal aggregates are more abundant within the basal and intermediate turns of the cochlea. Consistently, in the course of human degenerative tinnitus, the pathology of OHC in *ATG7* KO mice progresses from basal to apical turns. In degenerating OHC, the levels of prestin do not decrease massively, although prestin is rather dispersed and may not work properly. As expected, mitochondria are accumulated both at infra-nuclear and infra-cuticular levels within OHC. The accumulation also involves mitochondrial remnants within autophagosomes, which are aggregated as much as p62 protein [12] (Figure 17). The specific kind of mitochondrial alterations within these OHCs consists of a dismantling of the inner mitochondrial membrane before the rupture of the outer membrane, which is opposite to natural mitochondrial degradation during physiological mitochondrial turnover. Early in the process, mitochondrial alterations start with protruding mitochondrial cristae of mutant OHCs, which start to shorten, swell, deform, collapse, or disappear, which indicates reduced inner membrane surface area together with low-density mitochondrial mass. These degenerated mitochondria can be found within autophagosomes. Nonetheless, due to a deficit in the autophagy machinery, most altered mitochondria cannot be taken up by autophagosomes, and they appear partially enwrapped by the limiting autophagosomal membranes. This is likely to depend on the failure of the autophagy/mitophagy machinery in ATG7 KO, which impedes the membrane elongation process and is inadequate to clear damaged mitochondria. It is important to emphasize how this kind of degeneration differs from OHC apoptosis, which features cell shrinkage, membrane blebbing, karyorrhexis, and apoptotic bodies. The seminal manuscript by Zhou et al. [12] indicates that Atg7-dependent autophagy is required for OHC integrity, SC function and orientation, lateral motility, mitochondrial health, and synapse working with type II afferents. Considering the innumerous pathways involved in the autophagy process, it is important to emphasize how the activity of OHC selectively relies on the specific activity of Atg7-dependent autophagy machinery. In fact, when Atg7-dependent autophagy is defective, abnormal SC push/pull drive is likely to occur (due to impaired energy stores) instead of normal shearing. On the contrary, the loss of consistency in the SC protein structure may generate a stereocilia slant. Similarly, a defect in autophagy is likely to alter the electrochemical properties of OHC, leading to synaptopathy between OHC and type II afferents. The key role of autophagy in promoting OHC survival can also be inferred from its specific effects on the *stria vascularis* (Figure 19).

In fact, when hypoxia takes place in the marginal cells of the *stria vascularis*, autophagy is suppressed [66]. This leads to a decrease in the amount of ZO-1 protein, which is essential to form the tight junctions of the reticular lamina (RL, Figure 10). This mechanism may alter the key structures where SC is attached. Despite some conflicting reports about the role of autophagy in mediating ototoxicity produced by AGs, a recent manuscript by Zhang et al. [67] indicates that specific mitochondrial removal through mitophagy is essential to protect against AG-induced ototoxicity. In detail, when neomycin is administered, dramatic mitochondrial damage takes place, which is induced by a dysregulation of the interaction between the two fundamental autophagy proteins PINK1/Parkin. In these conditions, suppression of lysosomal degradation of mitochondria is concomitant [67]. In fact, neomycin inhibits the transcription of PINK1 and increases the expression of ATF3, which inhibits mitophagy. The relevance of autophagy in the course of ototoxicity induced by AGs is evidenced by the reversal of AGs ototoxicity by administering a pharmacological mitophagy activator such as deferiprone (DFP) or by suppressing the expression of USP30, which is a de-ubiquitinating enzyme toning down the removal of altered mitochondria.

Remarkably, mitophagy restoration rescues the selective damage induced by AG, mostly with OHC and partly with IHC, which replicates the site-specificity of cell damage in degenerative tinnitus [67]. These data are in line with previous data by He et al. and Yang et al. [55,68], who demonstrated that autophagy and selective mitophagy protect HC from AG-induced toxicity. Remarkably, autophagy sorts the same protective effects on HC survival both during AG ototoxicity, in the course of degenerative ototoxicity, and during aging [69]. Again, the classic mechanistic target of rapamycin complex 1 (mTORC1) inhibitor and autophagy activator, rapamycin, is able to rescue both degeneration- and AG-induced OHC loss [70]. It seems that autophagy is truly a master regulator of HC survival since its protective effects extend to the cell bodies within the Corti ganglion. This is evident in both ototoxic drugs and degenerative tinnitus. In fact, autophagy protects against ototoxic exposure to cadmium [71]. Similarly, cisplatin-induced HC loss is prevented by the autophagy inducer trehalose [72] in a way that is suppressed by pre-administering the autophagy inhibitor 3-methyl adenine (3-MA). In the course of age-related degeneration of the spiral ganglion cells, the autophagy activator rapamycin rescues dying neurons and preserves ganglion cells [73]. This is not surprising considering that a loss of OHC and IHC produces the loss of trophic targets of primary afferents coming from the spiral ganglion. This confirms the findings by Guo et al. [74], showing that autophagy sorts concomitant beneficial effects on HC and ganglion cells. This occurs following exposure to ototoxic drugs, noise-induced ototoxicity, and age-related cochlear degeneration. These data sum up one main concept of the present review concerning the fact that, despite apparent divergent factors in producing tinnitus and hypoacusia, a common convergent mechanism consisting of altered cell clearing pathways within OHC SC exists.

The key question concerns the molecular mechanisms through which autophagy exerts such a powerful beneficial effect. A potential explanation provided by Liu et al. [73] indicates that a loss of autophagy reduces both the motility and stiffness of OHC SC. Both phenomena lead to alterations in MET activity, which was explained at the beginning of this review. In fact, the loss of SC motility reduces or reverts the dragging of the endolymph, while the loss of SC stiffness (SC slanting) alters radial shearing. In detail, the authors analyzed the pattern of age-related changes in specific genes by using RNA-sequencing transcriptomic analysis of both IHC and OHC and made an elegant correlation with cell structure and mechanical and electrical properties. The seminal changes involved oxidative stress and autophagy, which were affected by specific genes such as superoxide dismutase 1 (*SOD1*), *Sirtuin 6* (*Sirt*6), which promotes autophagy by interacting with *ULK1* [75,76], and chromobox protein homolog 3 (*CBX3*), which is involved in DNA repair. A number of genes were altered to a lesser extent, mostly within OHC, and most of them were related to autophagy, which lends substance to the concept promoted by Liu in the manuscript titled “Autophagy: A Novel Horizon for Hair Cell Protection” [77]. In fact, degenerative alterations of OHC occur following decreased expression of autophagy-related genes. This altered expression pattern is manifested through specific morphological changes that early affect OHC SC. These anatomical features of SC degeneration are likely to reflect an altered composition of SC due to an impairment of autophagy-dependent protein turnover, which affects SC biosynthesis and elongation. The property of keeping steady the length of SC requires a balance in actin protein turnover, which determines actin capping and actin severing [73,78]. The turnover of actin is seminal for the functional micro-anatomy of SC. In fact, the hair bundle in each HC is composed of roughly 100 SC with progressive length and width [79]. Despite being apparently simple, the architecture of HC SC is complex to maintain effective MET activity. Such an architecture is mainly shaped by actin composition, both shaping SC and anchoring its roots to the cuticular plate. In this context, actin interacts with a number of actin-binding proteins (ABPs) to cross-link actin filaments into specific topologies, as well as control actin filament growth, severing, and capping. These processes are critical for sensory transduction and are all disrupted in inherited disorders of hearing in humans [79]. Several of these actin-interacting proteins with capping activity occur in OHC SC [80]. These proteins, along with myosin interacting proteins, undergo a marked suppression during SC degeneration [73,81,82,83]. The turnover of proteins involved in actin capping and actin severing is markedly affected by autophagy status and may lead to SC abnormalities. The turnover of critical proteins regulating SC motility and stiffness is commonly affected during OHC degeneration due to autophagy impairment. 

## 9. The Genetics of Hearing Loss and Tinnitus May Include a Role of Autophagy and Autophagy Activators

Although inherited conditions caused by roughly 30 genes are causative for only 5–10% of adult-onset tinnitus and hearing loss, the specific proteins coded by these genes are relevant to understanding which pathways are likely to be relevant in sporadic conditions. For instance, a specific inherited condition characterized by tinnitus and hearing loss in humans is produced by mutations in the gene coding for OSBPL2 (oxysterol binding protein-like 2; MIM606731), so-called autosomal dominant hearing loss (DFNA67). Subjects affected by OSBPL2 mutations develop tinnitus and progress towards hearing loss between ages 5 and 40. Hearing loss starts with tones at high frequencies and progresses to lower frequencies over time [84]. Remarkably, when the *OSBPL2* gene is knocked out in mice, hearing loss does not occur, while a loss of hearing occurs when the mutated OSBPL2 protein is expressed following autosomal dominant mutations. In fact, in this condition, the mutant proteins are not cleared from the cell and lead to aggregates, which impair the autophagy machinery. Thus, the real mechanism leading to auditory impairment in patients affected by the *OSBPL2* gene has been questioned. In fact, these patients develop hypercholesterolemia along with cochlear hair cell loss, starting with morphological abnormalities of the SC. A number of hypotheses were formulated to explain how hypercholesterolemia may induce tinnitus and loss of hearing. Recent evidence clearly shows how OSBPL2 is not essential for hearing. It is rather the accumulation of the protein OSBPL2 that leads to the engulfment of the autophagolysosomal system within OHC, which alters the physiology of OHC, leading to tinnitus and deafness. In fact, these autophagy-dependent symptoms can be rescued by administering the mTOR inhibitor and autophagy activator rapamycin, which improve hearing and erase tinnitus in subjects affected by DFNA67 [84]. It is impressive how a quite ubiquitous protein, when mutated, apart from altering cholesterol metabolism, leads specifically to hearing loss and tinnitus. This is in line with the seminal role that is exerted by effective autophagy within OHC. In fact, the massive energy demand engages these cells to produce a high amount of oxidative by-products, which alter autophagy substrates. In these conditions, a further increase in autophagy demands severe deleterious effects since OHC cannot cope with the further increase in this cell-clearing activity. 

Autophagy analysis of familial cases of tinnitus enabled the identification of several genes associated with sensorineural tinnitus, hearing loss (SNHL), and Meniére disease (MD). In most cases, these genes possess specific functions within the inner ear. For instance, in MD, the most frequently mutated genes are *OTOG*, *MYO7A*, and *TECTA* [85]. According to the crucial role of the tectorial membrane and its relationship with the reticular lamina and OHC in producing tinnitus and hearing loss, these genes alter these structures. In fact, *OTOG* encodes for otogelin, a protein that belongs to the TM, where it works to produce the composition of glycoproteins known as epithelial mucins. The occurrence of a properly structured otogelin is key to providing the stability and physiology of TM. The altered structure of TM due to a mutation in the otogelin impairs the relationship between TM and OHC SC, which in turn may produce TM dimpling or tenting, causing tinnitus and hearing loss, as previously reported in the present review. In fact, the MET coupling of OHC is impaired, and the contribution of OHC may reverse the auditory stimulation as described above. This is due to a loss of stability in the TM, which is no longer shearing stereocilia up to the level needed to open the cation channels upon traction of the tip link. The abnormal transduction in the channel complex alters the electrical effects due to radial shearing. Indeed, the relevance of otogelin is expected to apply to the vestibular system since Meniere syndrome occurs following this mutation. In fact, otogelin is also present in the cupule of the ampullary crests of the semicircular canals and within the otolithic membrane, which occurs within the otolithic macules of the utriculus and sacculus.

When studying a number of non-related families with MD, pathogenic or unknown-significance variants were reported in the *OTOG* gene [86]. The functional effects of variants in this gene could be better demonstrated in animal models than in vivo in patients. Mutant mice that lack otogelin or otogelin-like (OTOGL) proteins exhibit significant dysfunction of the OHC. Despite some MET remaining, there is a significant absence of acoustic distortion products since the anchorage of OHC SC to TM is altered. In detail, the specific alterations found in OTOG−/− mice consist of the loss of horizontal top connectors (side links) between the SC of OHCs. These fibrous links are critical to connecting neighboring stereocilia, and they are a key structural component of TM-attachment crowns that connect the tallest stereocilia of OHC SC to the TM [87].

Another gene, the *MYO7A* gene, codes an unconventional myosin (myosin VIIA), which is expressed within the SC of HC both in the cochlea and vestibular system [88]. 

Myosin VIIA is essential for the early development of the hair bundle of cochlear and vestibular organs, and, together with myosin 1C, it acts as an engine that keeps the structure of the tip link tense, allowing MET [89].

Ultimately, these abnormalities can lead to the development of hearing loss and/or vestibular dysfunction [90].

Moreover, in different families with MD, co-segregation has been reported in several novel and rare variants in the *MYO7A* gene with other genes involved in the MET complex and the top- and side-links of HCs. These include cadherin-23 (CDH23) and protocadherin-15 (PCDH15), two calcium-dependent cell adhesion proteins that participate in the structure of the tip-links, as well as adhesion G protein-coupled receptor V1 (ADGRV1), a protein of the ankle-link [91]. 

Moreover, rare missense variants and structural variants in synaptic genes, including *ANK2*, *TSC2*, and *AKAP9*, have been identified both in MD patients and in a cohort of patients with severe tinnitus [92]. 

Mutations in detoxifying and antioxidant phase I and II enzymes can alter their functions, resulting in excessive ROS production. Among them, mutations in cytochrome P450 superfamily enzymes (CYPs), such as CYP2B6 [93] and CYP1A1 [94], were associated with a greater predisposition to MD and tinnitus with presbyacusis, respectively.

## 10. The Multiple Significance of Findings Obtained Following Ototoxic Drugs

Several drugs produce cochlear–vestibular symptoms, including hearing loss, aural fullness, dizziness, vertigo, and tinnitus; among these, the strongest are platinum-derived chemotherapeutic agents (e.g., cisplatin) and some antibiotics (e.g., AG) [95].

Apart from the marked involvement of autophagy in the molecular mechanisms of AGs-induced toxicity (Figure 17 and Figure 18), which were previously reviewed in the paragraph about autophagy, ototoxic drugs also possess some specific effects that need to be reported.

Cisplatin administration induces sensorineural hearing loss with stria vascularis (Figure 19) and Corti’s spiral ganglion injury, along with hair cell death. The molecular pathways underlying this detrimental mechanism include both transport channels for cisplatin uptake and ROS production [96]. Previously, in the review, the involvement of autophagy in cisplatin toxicity was greatly emphasized. Nonetheless, cisplatin possesses a number of effects on the inner ear.

Cisplatin has a number of targets: (i) marginal cells of the stria vascularis, (ii) OHCs, and (iii) mtDNA in all cell types. 

**Figure 20:**
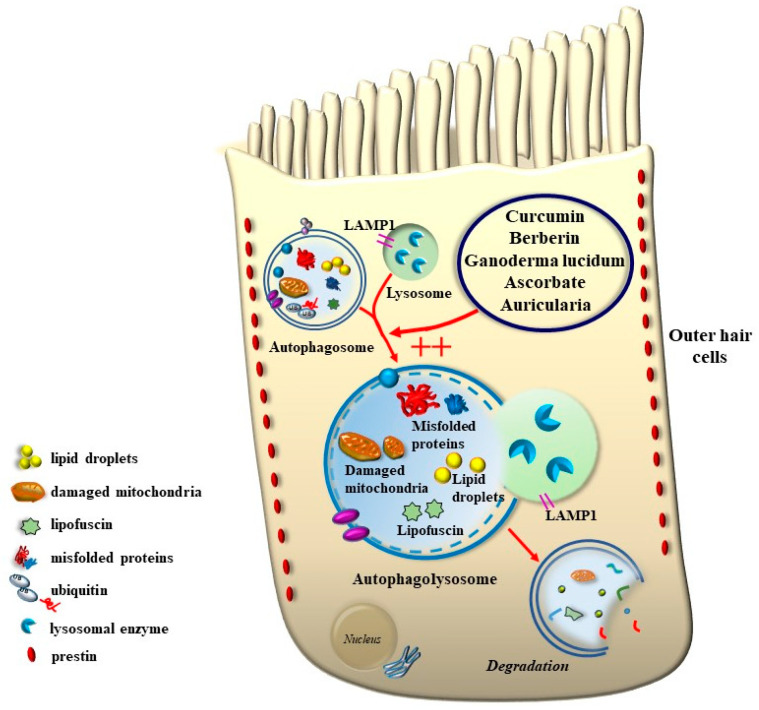
A number of phytochemicals may have a beneficial effect on the neuropathology of tinnitus. Curcumin, Berberine, and Ganoderma lucidum share the property of enhancing autophagy activity, which allows them to counteract early and late cytopathology occurring within OHC (and, to a lesser extent, other cells of the inner ear) during tinnitus. This representative picture focuses on the common properties of lysosomes digesting proteins, lipids, and mitochondria in order to sustain the high energy demand and ROS-induced damage that take place in these active cells.

Within the stria vascularis, cisplatin operates the breakdown of marginal cells, which impairs the number of active Na^+^/K^+^/2Cl-co-transporters. This suppresses the endolymphatic potential and endolymphatic homeostasis, which are responsible for the physiological function of IHC and OHC. When marginal cells are distorted, a significant amount of damage-associated molecular patterns (DAMPs), including those molecules activating channels TPA1 of Hensen’s cells, is released in the endolymph (Figure 19). These include mtDNA, extracellular genomic DNA, and heat shock proteins, which produce a marked alteration in the inflammatory process. In fact, these compounds bind toll-like receptors (TLRs), including TPA1, placed on both Hensen’s cells and the HC plasma membrane [97]. In fact, TLR-4 may bind the MD2 ligand and other DAMPs or inflammatory proteins, such as lipopolysaccharide (LPS). The activation promotes the TIRAP/MyD88-TAK1 pathway, which is paced downstream in the cell [98]. Concomitantly, NF-kB translocates into the nucleus to express a number of cytokines (e.g., TNF-α, IL-1β, and IL-6), which are released in the endolymph to expand inflammation and increase ROS production [99], which adds to the damage to OHCs (Figure 20). 

In this way, ototoxicity occurs, which manifests more as cochlear than vestibular damage, which is irreversible [96,100].

Cisplatin induces damage to mitochondria. At this level, it binds mtDNA, forming chemical adducts that hinder mtDNA replication and inhibit mtDNA transcription, thereby suppressing mitochondrial protein levels and producing misfolding proteins, which produce mitochondrial dysfunction [101]. Among ototoxic drugs, AGs are very common antibiotics widely used to treat Gram-negative bacterial infections. Ototoxic AGs such as dihydrostreptomycin, kanamycin, neomycin, amikacin, and tobramycin overlap partially with vestibulotoxic AGs (streptomycin, gentamicin, tobramycin, and sisomicin) [55,95,102,103]. Independently of the specific ototoxic or vestibular toxicity, AGs enter sensory HC; in the cochlea, they affect both IHC and OHC, while in the vestibule, both Type-I and Type-II HCs are impacted. 

Within the cochlea, these drugs augment ROS mostly in the basal turn to spread later towards the apex of the spiral channel. This is why lower tones are lost only upon repeated drug exposure. There is a remarkable gradient between the placement of OHC in the cochlea and the amount of ROS and cell death that are produced upon drug exposure [102].

## 11. Potential Benefits of Nutraceuticals 

**Figure 21:**
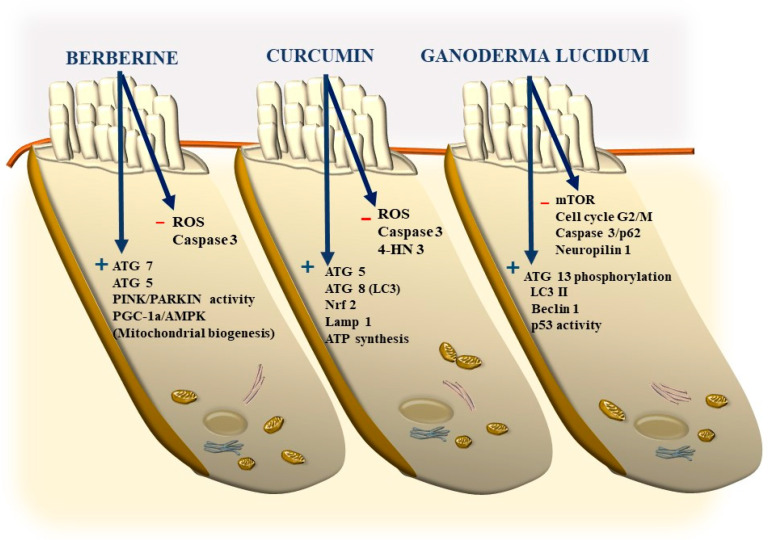
Some paradigms about the effects of berberine curcumin and Ganoderma lucidum. The cartoon shows some details that are key in counteracting the onset of tinnitus. All phytochemicals in the cartoon share the ability to elevate the number of key autophagy genes and proteins, such as Atg7, Atg5, and Atg13. These proteins are key to the onset of tinnitus since their genetic removal, specifically within OHC, generates experimental tinnitus. Similarly, all compounds act on key proteins involved in mitochondrial turnover, such as PINK 1 and parkin, and mitochondrial biogenesis, such as peroxisome proliferator-activated receptor (PPAR)-γ coactivator-1α (PGC-1α). Most autophagy proteins, and specifically those proteins acting to commit the autophagosomes, such as beclin1 and LC3, are increased as well.

**Figure 22:**
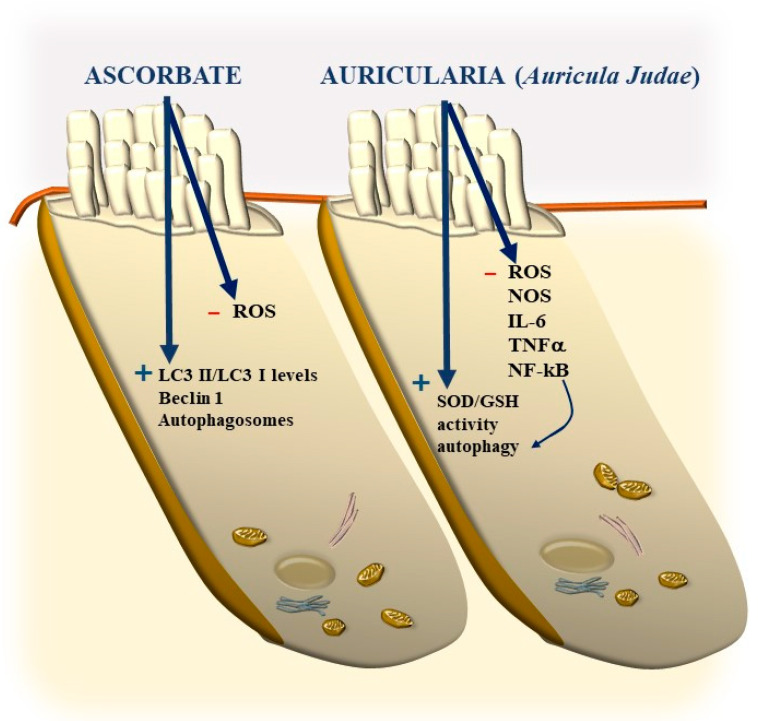
Ascorbate and Auricularia are synergic in counteracting molecular alterations in tinnitus. The cartoon emphasizes the concomitant stimulation of autophagy components and the decrease in ROS, which is produced by these compounds.

The marked role of autophagy in causing toxicity and degeneration of HC, leading to tinnitus, hypoacusia, and vestibular symptoms, calls for a protective effect of autophagy-inducing molecules (Figure 20, Figure 21 and Figure 22).

In fact, when analyzing the cytopathology of HCs following autophagy inhibition, the pathological findings overlap considerably with those obtained in degenerative tinnitus and hearing loss. In detail, when the autophagy-stimulating protein ATG5 is suppressed, OHC develops neuronal inclusions staining for p62 and ubiquitin along with abnormal organelles, including mitochondria, which indicates how basal autophagy is essential for HCs [61]. This is disrupted in all conditions leading to tinnitus, including the effects of ototoxic AGs [71,104]. The essential role of autophagy in the inner ear extends to the vestibular system, where the autophagy initiator Atg4b is needed for the development of otoconia [105]. In fact, a decrease in autophagy activity impairs the formation and aggregation of otoconial proteins, which leads to altered equilibrium. Altogether, these findings suggest the relevance of autophagy to hair cells, endolymph composition, and sensory membranes (otoconial, tectorial, and cupular membranes), which are typical of the inner ear. This scenario calls for a drug combination aimed at promoting autophagy, acting in multiple steps.

Autophagy activators were already mentioned in the course of the review, although most of these molecules—despite being very effective—have significant toxicity and a number of side effects. This is the case of rapamycin, which is unlikely to be administered for prolonged time intervals. In recent years, natural dietary compounds named phytochemicals have been intensely studied due to their marked protective effects on cell viability. Most of these compounds have significant pro-autophagy activity along with antioxidant and anti-inflammatory properties and were tested in a number of disorders and organs. A number of phytochemicals may help to reduce oxidative damage in the auditory system, mitigating inflammation and enhancing blood flow in the affected area. 

So far, a number of compounds have been tested, aiming to protect against HC death and hearing loss based on their antioxidant effects. In mouse models, treatments with alpha-lipoic acid (ALA) or red ginseng attenuate oxidative DNA lesions due to noise exposure. Coenzyme Q10 (CoQ10), a component of the mitochondrial respiratory chain, acts as a ROS scavenger and prevents oxidative stress-induced apoptosis. Also, vitamins, including vitamins A, B_12_, C, and E, can protect the inner ear [106].

Phytochemicals add to the rough concept of generic antioxidant activity since some of these compounds may produce a precise beneficial effect counteracting specific detrimental phenomena, which were described in the course of OHC degeneration in patients affected by tinnitus and presbyacusis. Among these natural compounds, special emphasis concerns berberine and curcumin, which, apart from possessing antioxidant activity, exert specific molecular effects to counteract those biochemical steps that are altered early during HC degeneration.

### 11.1. Berberine 

This is an isoquinoline alkaloid found in many medicinal plants, including Berberis (*B. vulgaris, B. aquifolium*, and *B. aristata*), *Hydrastis canadensis*, *Phellodendron amurense*, and *Coptis chinensis* [107]. Berberine has been widely used based on its anti-inflammatory and anti-oxidative properties, which were extensively observed in different disorders such as cardiovascular disease, diabetes, cancer, neurodegenerative disease, and others [108,109].

The protective effect of berberine on the inner ear at the level of HC is now clearly established in various experimental models (Figure 21).

In organotypic cell culture from mouse cochlea, pre-treatment with berberine chloride prevents cochlear HC loss and SC degeneration induced by AG drugs such as amikacin, kanamycin, and gentamicin. In berberine-treated culture, ROS accumulation is inhibited along with DNA fragmentation and loss of mitochondrial membrane potential [110].

In vivo experiments confirm these observations. Recently, Kilic et al. [111] described the efficacy of berberine on noise-induced ROS accumulation in rats. Acoustic trauma (rats exposed to white noise for 12 h; 4 kHz, 110 dB) causes severe histopathological impairment of cochlear structures along with elevation of 8-hydroxy-2-deoxyguanosine (8-OHdG), a marker of oxidative DNA damage. Berberine partially reverses the detrimental effects of acoustic trauma. Rats with acoustic trauma who are treated with 100 mg/kg of berberine for five consecutive days exhibit minimal histopathological changes and a reduction in the 8-OHdG expression in the spiral ganglion of Corti and OHCs [111].

Zhao and colleagues (2021) compared free berberine with berberine nanoparticle activity in guinea pigs exposed to broad-band white noise (4 h, 6.3–20 kHz at 120 dB). The free berberine group produces lower MDA levels and higher SOD activity compared with non-berberine-treated controls, indicating that berberine has antioxidant effects. Interestingly, berberine nanoparticles have a stronger effect on modulating MDA levels and SOD activity. Being able to cross the blood–labyrinth barrier (BLB), nanoparticles can directly accumulate in HCs to fulfill their protective role. Guinea pigs treated with berberine nanoparticles show morphological integrity of OHCs and improved hearing [112].

Mitochondria are one of the primary targets for berberine, which can modulate mitochondrial activity, mitophagy, and mitochondrial biogenesis [113].

In vitro assays have observed that berberine, encapsulated in lipid nanoparticles (LNPs), accumulates in mitochondria and acts on mitochondrial respiratory chain complex I, resulting in increased intracellular ATP production. Berberine-LNPs enhance the expression of mitochondrial ubiquitin ligase, supporting mitochondrial quality control [114]. Moreover, berberine increases mitochondrial membrane potential and induces mitophagy through the activation of the PINK1-Parkin pathway [96].

Berberine exerts its protective effects by also targeting peroxisome proliferator-activated receptor (PPAR)-γ coactivator-1α (PGC-1α). PGC-1α is a transcriptional regulator responsible for controlling mitochondrial biogenesis and function. In mice, the overexpression of PGC-1α leads to an enhancement in mitochondrial biogenesis and an increase in the expression of mitochondrial genes. Conversely, in mice lacking PGC-1α, a decrease in oxidative metabolism and mitochondrial content is observed. 

Berberine promotes mitochondrial biogenesis and increases energy production in an AMP-activated protein kinase (AMPK)-dependent manner. In fact, berberine increases AMPK and PGC-1α protein levels [115].

Most of these effects of berberine are likely to be indirectly induced by its strong autophagy promotion. In fact, the tinnitus and hearing loss produced by knocking down ATG7-dependent autophagy, specifically within OHCs, are reverted by barbering, which increases Atg7 along with Atg5, making it interesting to be tested to antagonize the pathology of OHC, Wang et al. (2017) (Figure 18 and Figure 19).

Taken together, these results indicate that berberine has the potential to protect HCs against oxidative damage by suppressing ROS production, improving mitochondrial functions, and promoting mitophagy and mitochondrial energy metabolism. These findings highlight berberine as a therapeutic strategy to safeguard mitochondria and mitigate the harmful effects of oxidative stress.

### 11.2. Curcumin 

This is an active component of turmeric, *Curcuma longa*, which acts on multiple biological targets (Figure 21) and molecular pathways involved in aging, inflammation, and oxidative stress. Therefore, curcumin is widely used as a therapeutic compound against several diseases [116]. In the last decades, some studies have analyzed the ability of curcumin to counteract drug-induced ototoxicity.

When tested against ototoxicity induced by paclitaxel, a taxane plant product largely used as an anticancer agent, curcumin prevents damage at various sites, including OHC, stria vascularis, and spiral limbus. At this level, curcumin decreases cytoplasmic vacuolization and prevents the atrophy of intermediate cells within the stria vascularis. The protective effects of curcumin against paclitaxel-induced ototoxicity were attributed to a number of effects [117].

Similarly, during cisplatin-induced ototoxicity, Soyalıç et al. [118] found that curcumin suppresses the decrease in hearing while preventing oxidative stress both at the level of OHCs, spiral ganglion cells, and stria vascularis [118].

Curcumin reverses the toxicity induced by gentamicin and sodium salicylates. These effects are accompanied by preventing the depletion of antioxidant defense and suppressing the increase in lipid peroxidation (MDA) levels. A number of downstream effects are described as protective against OHC SC loss following curcumin administration. This is the case of altered levels of NF-kB, caspase-3, and Bcl-2 expression [119].

Curcumin may also prevent noise-induced hearing loss. In fact, curcumin (100 mg/kg) attenuates noise-induced hearing loss, which follows a 1-h daily exposure to 90 dB for five days. Again, such an effect is concomitant with reduced levels of 4-HNE and NF-kB activation [120].

In particular, the suppression of lipid peroxidation was associated with protective effects against disruption of ZO-1-rich gap junctions. Moreover, curcumin prevents the nuclear translocation of the active subunit of NF-kB in the cochlear spiral ligament, probably through inhibited degradation of inhibitor kB (IkB), preserving the endo-cochlear potential [120].

Curcumin is a powerful activator of nuclear factor erythroid 2-related factor 2 (Nrf2), a prominent transcription factor responsible for regulating the expression of genes involved in antioxidant and detoxification processes. Once activated, Nrf2 translocates into the nucleus, where it binds to specific DNA sequences, the antioxidant response elements (AREs), and interacts with other proteins, thereby regulating the transcription of a wide range of genes. These genes encompass various antioxidant enzymes, detoxifying enzymes, and cytoprotective proteins, including heme oxygenase-1 (HO-1), NAD(P)H, quinone oxidoreductase 1 (NQO1), GPx, catalase, and SOD.

Curcumin is likely to increase the expression of Nrf2 within the Corti organ, which is likely to produce anti-inflammatory events. For instance, cisplatin administration within HEI-OC1 cells transfected with wild-type Nrf2 is less effective in secreting TNF-α, IL-1β, and IL-6. Conversely, those cells expressing a dominant-negative mutant of Nrf2 (dn-Nrf2) show an increase in TNF-α and IL-1β levels. The inhibition of pro-inflammatory cytokines is attributed to the action of Nrf2 on the NF-kB pathway [121].

In HEI-OC1 cells, curcumin decreases oxidative stress, senescence, and apoptosis induced by H_2_O_2_. In fact, curcumin treatment reduced ROS and senescence-associated β-galactosidase (SA-β-gal) levels as well as the expression of caspase-3. Moreover, curcumin restores altered mitochondrial membranes, enabling ATP synthesis and providing a protective effect on mitochondria. Again, these effects are likely to be mediated by Nrf2, since curcumin is no longer able to reduce ROS levels and alter mitochondrial membrane potential when Nrf2 is suppressed by siRNA [104].

All these reports suggest that a number of protective effects of curcumin on auditory HCs could be mediated by the Nrf2 pathway. Interestingly, the Nrf2 pathway has a powerful cross-talk with the autophagy machinery [122]. Indeed, recent findings correlate the effects of curcumin on the Nrf2 pathway with its properties as an autophagy inducer since autophagy activation appears to be essential in regulating the effects of the Nrf2 pathway [123]. For instance, a recent study indicates that protective effects induced by curcumin against oxidative stress produced by doxorubicin depend on the autophagy regulation of Keap1–Nrf2–ARE signaling pathways [123]. In fact, most of the effects exerted by curcumin are produced under the powerful regulation of the autophagy machinery. In fact, the effects induced by curcumin as an autophagy inducer may explain most of the downstream effects described above. For instance, curcumin promotes the synthesis of Atg5 and Atg7, and this is postulated to underlie most of its activity in counteracting tinnitus [124].

In the same experimental condition, curcumin upregulates the level of microtubule-associated protein 1 light chain 3-II (LC3-II), the number of autophagosomes, and the degradation of p62. The powerful effects of curcumin as an autophagy activator are also mediated by an increase in beclin1 and the inhibition of the phosphatidylinositol 3-kinase (PtdIns3K)-AKT-mechanistic target of the rapamycin (mTOR) signaling pathway [124]. Thus, the prominent effect of curcumin as a potential candidate to counteract degeneration of OHC covers a number of actions, which are mostly related to the stimulation of autophagy. In fact, even mitochondrial damage, which is constantly described at the infra-cuticular and infra-nuclear levels within OHC, is likely to benefit from curcumin since a number of genes and proteins promoting mitochondrial quality control are stimulated by curcumin administration [125]. These effects are considered to produce most of the beneficial effects of curcumin, as well as inhibiting the amount of ROS.

## 12. Conclusions

A number of factors and mechanisms have been claimed to be responsible for the onset of tinnitus. Nonetheless, long-lasting chronic peripheral tinnitus is constantly accompanied by early degeneration of OHC in the Corti’s organ. Such an alteration starts with a loss of OHC SC and progresses towards OHC cell loss. Most cases of chronic peripheral tinnitus are accompanied by concomitant hearing loss. The present review is designed to advance our awareness of the connection between neuropathology, which manifests in the Corti’s organ, and the occurrence of tinnitus as a clinical symptom. Thus, the present review analyzes the main mechanisms that are responsible for the generation of sound by OHC and how these altered mechanisms may induce phantom noise during tinnitus. In fact, amongst a number of mechanisms, the activity of OHC is seminal for noise generation during this symptom. Therefore, in the present review, the mechanisms operating within the Corti’s organ under the influence of OHC were discussed first before analyzing the cell pathology of tinnitus. In the second part, the specific metabolic pathways that are involved in the disorder were discussed. Thus, the review indicates the main subcellular pathology of OHC SC and the whole OHC. This includes the accumulation of mitochondria at sub-cuticular and sub-nuclear levels, the accumulation of misfolded proteins, and the accumulation of lipid droplets within OHC. All these changes are reminiscent of neurodegeneration. In fact, the very same pathology is transferred from OHC to neurons within the Corti ganglion, where neuronal inclusions in patients with tinnitus can be found. This configures chronic persistent tinnitus and prebyacusis as a selective neurodegenerative disorder affecting neural cells within the inner ear. In this progressive degeneration, some metabolic pathways are relevant. This is the case of autophagy, which is seminal for the onset of neurodegeneration within the CNS and plays a strong protective effect at the level of OHC. In detail, the present review provides converging experimental evidence from specific genetic and toxic models that, among various autophagy steps, the OHC is strongly dependent on Atg7-mediated autophagy. This explains why the conditional focal deletion of Atg7 produces a loss of OHC, which occurs following specific steps that mimic the neuropathology of tinnitus. Even in human disorders where tinnitus can be measured, the neuropathology is overlapping and autophagy is impaired. Strategies to enhance autophagy may be harmful due to side effects. This is the case of rapamycin, which needs to be carefully monitored (between 5 and 15 ng/mL) to avoid serious toxicity. Novel compounds shaped by the evolution of living organisms occur in various plants, and they are defined as phytochemicals. The review provides evidence showing a significant improvement of autophagy within the inner ear and OHC by specific phytochemicals such as curcumin and berberine. This shed light on novel therapeutic approaches to improve the time course of tinnitus and counteract the concomitant presbyacusis.

## Abbreviations

AA: arachidonic acid; ABPs: actin-binding proteins; ADGRV1: adhesion G protein-coupled receptor V1; AGs: aminoglycosides; ALA: alpha-lipoic acid; AREs: antioxidant response elements; ATF3: Activating Transcription Factor 3; ATG: autophagy; BM: basilar membrane; BLB: blood–labyrinth barrier; CBX3: chromobox protein homolog 3; CCCP: carbonyl cyanide m-chloro-phenyl-hydrazone; CDH23: cadherin-23; CoQ10: coenzyme Q10; CYPs: cytochrome P450 superfamily enzymes: CNS: central nervous system; DAMPs: damaged associated molecular patterns; DFP: deferipone; DFNA67: autosomal dominant hearing loss; FOXG1: Forkhead box G1; GPI: glycosyl-phosphatidyl-inositol; GPx: glutathione peroxidase; GR: glutathione reductase; GSH: reduced glutathione; GST: glutathione S-transferase; HC: hair cells; HEI-OC1: House Ear Institute-Organ of Corti 1; 4-HNE 4-hydroxynonenal; HO-1: heme oxygenase-1; H_2_O_2_: hydrogen peroxide; IHC: inner hair cells; IMM: inner mitochondrial membrane; LC3: microtubule-associated protein 1 light chain 3; LLP: long-lived proteins; LPS: lipopolysaccharide; LNPs: lipid nanoparticles; 3-MA: 3-methyladenine; MD: Menière disease; MDA: malondialdehyde; MET: mechano-electrical transduction; MPO: myeloperoxidase; mTORC1: mechanistic target of rapamycin complex 1; NATs: N-acetyltransferases; NF-kB: Nuclear factor kappa B; NO: nitric oxide; NOs: nitric oxide synthases; Nrf2: nuclear factor erythroid 2-related factor 2; NQO1: quinone oxidoreductase 1; OH-: hydroxyl radical; 8-OHdG: 8-hydroxy-2-deoxyguanosine; OHC: outer hair cells; OMM: outer mitochondrial membrane; OSBPL2: oxysterol binding protein like 2; PARL: Presenilin-associated rhomboid-like protease; PCDH15: protocadherin-15; PGC-1α: coactivator-1α; PINK1: PTEN-induced kinase 1; PON: paraoxonase-1; PPAR: peroxisome proliferator-activated receptor; PRKN: Parkin; PtdIns3K: phosphatidyl inositol 3 kinase; RL: reticular lamina; ROS: reactive oxygen species; SC: stereocilia; Sirt6: Sirtuin 6; SH: sulfhydryl group; SLC: solute carrier family; SOD: superoxide dismutase; SNHL: sensorineural tinnitus, hearing loss; SQSTM1: sequestosome 1; TAS: total antioxidant status; TM: tectorial membrane; TRL-4: toll-like receptor-4; TRX: thioredoxin; TRPA1: Transient Receptor Potential Cation Channel A1; UCPs: uncoupled proteins; ZO1: Zonula Occludens 1.

## References

[1] Von Békésy G. (1956). Simplified model to demonstrate the energy flow and formation of traveling waves similar to those found in the cochlea. Proc. Natl. Acad. Sci. USA.

[2] Goutman J.D., Elgoyhen A.B., Gómez-Casati M.E. (2015). Cochlear hair cells: The sound-sensing machines. FEBS Lett..

[3] Fettiplace R. (2017). Hair Cell Transduction, Tuning, and Synaptic Transmission in the Mammalian Cochlea. Compr. Physiol..

[4] Zheng J., Shen W., He D.Z., Long K.B., Madison L.D., Dallos P. (2000). Prestin is the motor protein of cochlear outer hair cells. Nature.

[5] Liberman M.C., Gao J., He D.Z., Wu X., Jia S., Zuo J. (2002). Prestin is required for electromotility of the outer hair cell and for the cochlear amplifier. Nature.

[6] Ashmore J., Avan P., Brownell W.E., Dallos P., Dierkes K., Fettiplace R., Grosh K., Hackney C.M., Hudspeth A.J., Jülicher F. (2010). The remarkable cochlear amplifier. Hear. Res..

[7] Self T., Sobe T., Copeland N.G., Jenkins N.A., Avraham K.B., Steel K.P. (1999). Role of myosin VI in the differentiation of cochlear hair cells. Dev. Biol..

[8] Vijayakumar K.A., Cho G.W., Maharajan N., Jang C.H. (2022). A Review on Peripheral Tinnitus, Causes, and Treatments from the Perspective of Autophagy. Exp. Neurobiol..

[9] Matsumoto N., Kalinec F. (2005). Prestin-dependent and prestin-independent motility of guinea pig outer hair cells. Hear. Res..

[10] Ge J., Elferich J., Dehghani-Ghahnaviyeh S., Zhao Z., Meadows M., von Gersdorff H., Tajkhorshid E., Gouaux E. (2021). Molecular mechanism of prestin electromotive signal amplification. Cell.

[11] Guinan J.J. (2012). How are inner hair cells stimulated? Evidence for multiple mechanical drives. Hear. Res..

[12] Zhou H., Qian X., Xu N., Zhang S., Zhu G., Zhang Y., Liu D., Cheng C., Zhu X., Liu Y. (2020). Disruption of Atg7-dependent autophagy causes electromotility disturbances, outer hair cell loss, and deafness in mice. Cell Death Dis..

[13] Peixoto-Pinheiro B., Adel Y., Knipper M., Müller M., Löwenheim H. (2021). Auditory Threshold Variability in the SAMP8 Mouse Model of Age-Related Hearing Loss: Functional Loss and Phenotypic Change Precede Outer Hair Cell Loss. Front. Aging Neurosci..

[14] Lin X., Haller P., Bavi N., Faruk N., Perozo E., Sosnick T.R. (2023). Folding of Prestin’s Anion-Binding Site and the Mechanism of Outer Hair Cell Electromotility. bioRxiv.

[15] Burwood G., He W.X., Fridberger A., Ren T.Y., Nuttall A.L. (2022). Outer hair cell driven reticular lamina mechanical distortion in living cochleae. Hear. Res..

[16] Nuttall A.L., Fridberger A. (2012). Instrumentation for studies of cochlear mechanics: From von Bekesy forward. Hear. Res..

[17] Robles L., Ruggero M.A. (2001). Mechanics of the mammalian cochlea. Physiol. Rev..

[18] Chen F., Zha D., Fridberger A., Zheng J., Choudhury N., Jacques S.L., Wang R.K., Shi X., Nuttall A.L. (2011). A differentially amplified motion in the ear for near-threshold sound detection. Nat. Neurosci..

[19] Fallah E., Strimbu C.E., Olson E.S. (2019). Nonlinearity and amplification in cochlear responses to single and multi-tone stimuli. Hear. Res..

[20] Olson E.S., Strimbu C.E. (2020). Cochlear mechanics: New Insights from Vibrometry and Optical Coherence Tomography. Curr. Opin. Physiol..

[21] Braun M. (1996). Impediment of basilar membrane motion reduces overload protection but not threshold sensitivity: Evidence from clinical and experimental hydrops. Hear. Res..

[22] Fridberger A., Boutet de Monvel J., Ulfendahl M. (2002). Internal Shearing within the Hearing Organ Evoked by Basilar Membrane Motion. J. Neurosci..

[23] Fridberger A., Tomo I., Ulfendahl M., Boutet de Monvel J., Nuttall A. (2006). Stereociliary Vibration in the Guinea Pig Cochlea. Auditory Mechanics: Processes and Models.

[24] Fridberger A., Tomo I., Ulfendahl M., Boutet de Monvel J. (2006). Imaging hair cell transduction at the speed of sound: Dynamic behavior of mammalian stereocilia. Proc. Natl. Acad. Sci. USA.

[25] Vélez-Ortega A.C., Stepanyan R., Edelmann S.E., Torres-Gallego S., Park C., Marinkova D.A., Nowacki J.S., Sinha G.P., Frolenkov G.I. (2023). TRPA1 activation in non-sensory supporting cells contributes to regulation of cochlear sensitivity after acoustic trauma. Nat. Commun..

[26] Fechner F.P., Burgess B.J., Adams J.C., Liberman M.C., Nadol J.B. (1998). Dense innervation of Deiters’ and Hensen’s cells persists after chronic differentiations of guinea pig cochleas. J. Comp. Neurol..

[27] Wright C.G., Preston R.E. (1976). Efferent nerve fibers associated with the outermost supporting cells of the organ of Corti in the guinea pig. Acta Otolaryngol..

[28] Karavitaki K.D., Mountain D.C. (2007). Imaging electrically evoked micro-mechanical motion within the organ of Corti of the excised gerbil cochlea. Biophys. J..

[29] Hakizimana P., Fridberger A. (2021). Inner hair cell stereocilia are embedded in the tectorial membrane. Nat. Commun..

[30] Verpy E., Weil D., Leibovici M., Goodyear R.J., Hamard G., Houdon C., Lefèvre G.M., Hardelin J.P., Richardson G.P., Avan P. (2008). Stereocilin-deficient mice reveal the origin of cochlear waveform distortions. Nature.

[31] Strimbu C.E., Prasad S., Hakizimana P., Fridberger A. (2019). Control of hearing sensitivity by tectorial membrane calcium. Proc. Natl. Acad. Sci. USA.

[32] Manley G.A. (2000). Cochlear mechanisms from a phylogenetic viewpoint. Proc. Natl. Acad. Sci. USA.

[33] Zwaenepoel I., Mustapha M., Leibovici M., Verpy E., Goodyear R., Liu X.Z., Nouaille S., Nance W.E., Kanaan M., Avraham K.B. (2002). Otoancorin, an inner ear protein restricted to the interface between the apical surface of sensory epithelia and their overlying acellular gels, is defective in autosomal recessive deafness DFNB22. Proc. Natl. Acad. Sci. USA.

[34] Patuzzi R.B., Yates G.K., Johnstone B.M. (1989). Outer hair cell receptor current and sensorineural hearing loss. Hear. Res..

[35] Langguth B., Kreuzer P.M., Kleinjung T., De Ridder D. (2013). Tinnitus: Causes and clinical management. Lancet Neurol..

[36] Shore S.E., Roberts L.E., Langguth B. (2016). Maladaptive plasticity in tinnitus—Triggers, mechanisms and treatment. Nat. Rev. Neurol..

[37] Roberson D.W., Rubel E.W. (1994). Cell division in the gerbil cochlea after acoustic trauma. Am. J. Otol..

[38] Celik M., Koyuncu İ. (2018). A Comprehensive Study of Oxidative Stress in Tinnitus Patients. Indian J. Otolaryngol. Head Neck Surg..

[39] Hosseinzadeh A., Kamrava S.K., Moore B.C.J., Reiter R.J., Ghaznavi H., Kamali M., Mehrzadi S. (2019). Molecular Aspects of Melatonin Treatment in Tinnitus: A Review. Curr. Drug Targets.

[40] Ziegler E.A., Brieger J., Heinrich U.R., Mann W.J. (2004). Immunohistochemical localization of cyclooxygenase isoforms in the organ of Corti and the spiral ganglion cells of guinea pig cochlea. ORL J. Otorhinolaryngol. Relat. Spec..

[41] Jones D.P. (2008). Radical-free biology of oxidative stress. Am. J. Physiol. Cell Physiol..

[42] Harris C., Hansen J.M. (2012). Oxidative stress, thiols, and redox profiles. Methods Mol. Biol..

[43] Ramkumar V., Mukherjea D., Dhukhwa A., Rybak L.P. (2021). Oxidative Stress and Inflammation Caused by Cisplatin Ototoxicity. Antioxidants.

[44] Fetoni A.R., Paciello F., Rolesi R., Paludetti G., Troiani D. (2019). Targeting dysregulation of redox homeostasis in noise-induced hearing loss: Oxidative stress and ROS signaling. Free Radic. Biol. Med..

[45] Neri S., Mauceri B., Cilio D., Bordonaro F., Messina A., Malaguarnera M., Savastano M., Brescia G., Manci S., Celadini M. (2002). Tinnitus and oxidative stress in a selected series of elderly patients. Arch. Gerontol. Geriatr. Suppl..

[46] Neri S., Signorelli S., Pulvirenti D., Mauceri B., Cilio D., Bordonaro F., Abate G., Interlandi D., Misseri M., Ignaccolo L. (2006). Oxidative stress, nitric oxide, endothelial dysfunction and tinnitus. Free Radic. Res..

[47] Trevisani M., Siemens J., Materazzi S., Bautista D.M., Nassini R., Campi B., Imamachi N., Andrè E., Patacchini R., Cottrell G.S. (2007). 4-Hydroxynonenal, an endogenous aldehyde, causes pain and neurogenic inflammation through activation of the irritant receptor TRPA1. Proc. Natl. Acad. Sci. USA.

[48] Andersson D.A., Gentry C., Moss S., Bevan S. (2008). Transient receptor potential A1 is a sensory receptor for multiple products of oxidative stress. J. Neurosci..

[49] Bautista D.M., Pellegrino M., Tsunozaki M. (2013). TRPA1: A gatekeeper for inflammation. Annu. Rev. Physiol..

[50] Koç S., Akyüz S., Somuk B.T., Soyalic H., Yılmaz B., Taskin A., Bilinc H., Aksoy N. (2016). Paraoxonase Activity and Oxidative Status in Patients with Tinnitus. J. Audiol. Otol..

[51] Ekinci A., Kamasak K. (2020). Evaluation of serum prolidase enzyme activity and oxidative stress in patients with tinnitus. Braz. J. Otorhinolaryngol..

[52] Oh J., Youn C.K., Jun Y., Jo E.R., Cho S.I. (2020). Reduced mitophagy in the cochlea of aged C57BL/6J mice. Exp. Gerontol..

[53] Chen X., Wang Q., Li S., Li X.J., Yang W. (2022). Mitochondrial-Dependent and Independent Functions of PINK1. Front. Cell Dev. Biol..

[54] Kane L.A., Lazarou M., Fogel A.I., Li Y., Yamano K., Sarraf S.A., Banerjee S., Youle R.J. (2014). PINK1 Phosphorylates Ubiquitin to Activate Parkin E3 Ubiquitin Ligase Activity. J. Cell Biol..

[55] Yang Q., Zhou Y., Yin H., Li H., Zhou M., Sun G., Cao Z., Man R., Wang H., Li J. (2018). PINK1 Protects Against Gentamicin-Induced Sensory Hair Cell Damage: Possible Relation to Induction of Autophagy and Inhibition of p53 Signal Pathway. Front. Mol. Neurosci..

[56] Han H., Hu S., Hu Y., Liu D., Zhou J., Liu X., Ma X., Dong Y. (2023). Mitophagy in ototoxicity. Front. Cell. Neurosci..

[57] Cho S.I., Jo E.R., Song H. (2021). Mitophagy Impairment Aggravates Cisplatin-Induced Ototoxicity. BioMed Res. Int..

[58] Savas J.N. (2023). The cochlea is built to last a lifetime. Hear. Res..

[59] Mu Y.R., Zou S.Y., Li M., Ding Y.Y., Huang X., He Z.H., Kong W.J. (2023). Role and mechanism of FOXG1-related epigenetic modifications in cisplatin-induced hair cell damage. Front. Mol. Neurosci..

[60] Magarinos M., Pulido S., Aburto M.R., de Iriarte-Rodriguez R., Varela-Nieto I. (2017). Autophagy in the vertebrate inner ear. Front. Cell Dev. Biol..

[61] Fujimoto C., Iwasaki S., Urata S., Morishita H., Sakamaki Y., Fujioka M., Kondo K., Mizushima N., Yamasoba T. (2017). Autophagy is essential for hearing in mice. Cell Death Dis..

[62] Jongkamonwiwat N., Ramirez M.A., Edassery S., Wong A.C.Y., Yu J., Abbott T., Pak K., Ryan A.F., Savas J.N. (2020). Noise Exposures Causing Hearing Loss Generate Proteotoxic Stress and Activate the Proteostasis Network. Cell Rep..

[63] Wang W., Sun Y., Chen S., Zhou X., Wu X., Kong W., Kong W. (2015). Impaired unfolded protein response in the degeneration of cochlea cells in a mouse model of age-related hearing loss. Exp. Gerontol..

[64] Lee J.N., Kim S.G., Lim J.Y., Kim S.J., Choe S.K., Park R. (2015). Proteasome inhibitors induce auditory hair cell death through peroxisome dysfunction. Biochem. Biophys. Res. Commun..

[65] Wang H., Liu C., Mei X., Cao Y., Guo Z., Yuan Y., Zhao Z., Song C., Guo Y., Shen Z. (2017). Berberine attenuated pro-inflammatory factors and protect against neuronal damage via triggering oligodendrocyte autophagy in spinal cord injury. Oncotarget.

[66] Zhao Y., Huang S., Xie R., Liu J. (2023). Extracellular ATP accelerates cell death and decreases tight junction protein ZO-1 in hypoxic cochlear strial marginal cells in neonatal rats. Cell. Signal..

[67] Zhang Y., Fang Q., Wang H., Qi J., Sun S., Liao M., Wu Y., Hu Y., Jiang P., Cheng C. (2023). Increased mitophagy protects cochlear hair cells from aminoglycoside-induced damage. Autophagy.

[68] He Z., Guo L., Shu Y., Fang Q., Zhou H., Liu Y., Liu D., Lu L., Zhang X., Ding X. (2017). Autophagy protects auditory hair cells against neomycin-induced damage. Autophagy.

[69] Lv Z., Zhang Y., Cao H., Liu Q., Feng X., Yin H., Wang B. (2022). PIN1 protects auditory hair cells from senescence via autophagy. PeerJ.

[70] Xiong H., Pang J., Min X., Ye Y., Lai L., Zheng Y. (2022). miR-34a/ATG9A/TFEB Signaling Modulates Autophagy in Cochlear Hair Cells and Correlates with Age-related Hearing Loss. Neuroscience.

[71] Li Q., Wang L., Ji D., Yu W., Zhang Y., Xiang Y., Zhou C., Wang L., Deng P., Pi H. (2022). Metformin attenuates cadmium-induced degeneration of spiral ganglion neuron via restoring autophagic flux in primary culture. J. Inorg. Biochem..

[72] Li Z., Yao Q., Tian Y., Jiang Y., Xu M., Wang H., Xiong Y., Fang J., Lu W., Yu D. (2022). Trehalose protects against cisplatin-induced cochlear hair cell damage by activating TFEB-mediated autophagy. Biochem. Pharmacol..

[73] Liu H., Li F., Li X., Wu Q., Dai C. (2022). Rapamycin ameliorates age-related hearing loss in C57BL/6J mice by enhancing autophagy in the SGNs. Neurosci. Lett..

[74] Guo L., Cao W., Niu Y., He S., Chai R., Yang J. (2021). Autophagy Regulates the Survival of Hair Cells and Spiral Ganglion Neurons in Cases of Noise, Ototoxic Drug, and Age-Induced Sensorineural Hearing Loss. Front. Cell. Neurosci..

[75] Wu Z., Hong L., Luo G., Lu S., Li Y., Wang J., Zhang Y., Zhang L. (2022). SIRT6 promotes autophagy through direct interaction with ULK1 and competitive binding to PUMA. Genes Dis..

[76] Li C., Li S., Kong D.H., Meng X., Zong Z.H., Liu B.Q., Guan Y., Du Z.X., Wang H.Q. (2013). BAG3 is upregulated by c-Jun and stabilizes JunD. Biochim. Biophys Acta.

[77] Liu C., Zheng Z., Wang P., He S., He Y. (2021). Autophagy: A Novel Horizon for Hair Cell Protection. Neural Plast..

[78] Narayanan P., Chatterton P., Ikeda A., Ikeda S., Corey D.P., Ervasti J.M., Perrin B.J. (2015). Length regulation of mechanosensitive stereocilia depends on very slow actin dynamics and filament-severing proteins. Nat. Commun..

[79] Park J., Bird J.E. (2023). The actin cytoskeleton in hair bundle development and hearing loss. Hear. Res..

[80] Barr-Gillespie P.G. (2015). Assembly of hair bundles, an amazing problem for cell biology. Mol. Biol. Cell.

[81] Belyantseva I.A., Boger E.T., Naz S., Frolenkov G.I., Sellers J.R., Ahmed Z.M., Griffith A.J., Friedman T.B. (2005). Myosin-XVa is required for tip localization of whirlin and differential elongation of hair-cell stereocilia. Nat. Cell Biol..

[82] Stepanyan R., Frolenkov G.I. (2009). Fast adaptation and Ca^2+^ sensitivity of the mechanotransducer require myosin-XVa in inner but not outer cochlear hair cells. J. Neurosci..

[83] Manor U., Disanza A., Grati M., Andrade L., Lin H., Di Fiore P.P., Scita G., Kachar B. (2011). Regulation of stereocilia length by myosin XVa and whirlin depends on the actin-regulatory protein Eps8. Curr. Biol..

[84] Koh Y.I., Oh K.S., Kim J.A., Noh B., Choi H.J., Joo S.Y., Rim J.H., Kim H.Y., Kim D.Y., Yu S. (2022). *OSBPL2* mutations impair autophagy and lead to hearing loss, potentially remedied by rapamycin. Autophagy.

[85] Parra-Perez A.M., Lopez-Escamez J.A. (2023). Types of Inheritance and Genes Associated with Familial Meniere Disease. J. Assoc. Res. Otolaryngol..

[86] Roman-Naranjo P., Gallego-Martinez A., Soto-Varela A., Aran I., Moleon M.D.C., Espinosa-Sanchez J.M., Amor-Dorado J.C., Batuecas-Caletrio A., Perez-Vazquez P., Lopez-Escamez J.A. (2020). Burden of Rare Variants in the *OTOG* Gene in Familial Meniere’s Disease. Ear Hear..

[87] Avan P., Le Gal S., Michel V., Dupont T., Hardelin J.P., Petit C., Verpy E. (2019). Otogelin, otogelin-like, and stereocilin form links connecting outer hair cell stereocilia to each other and the tectorial membrane. Proc. Natl. Acad. Sci. USA.

[88] Gan N.S., Oziębło D., Skarżyński H., Ołdak M. (2023). Monogenic Causes of Low-Frequency Non-Syndromic Hearing Loss. Audiol. Neurootol..

[89] Li S., Mecca A., Kim J., Caprara G.A., Wagner E.L., Du T.T., Petrov L., Xu W., Cui R., Rebustini I.T. (2020). Myosin-VIIa is expressed in multiple isoforms and essential for tensioning the hair cell mechano-transduction complex. Nat. Commun..

[90] Roman-Naranjo P., Moleon M.D.C., Aran I., Escalera-Balsera A., Soto-Varela A., Bächinger D., Gomez-Fiñana M., Eckhard A.H., Lopez-Escamez J.A. (2021). Rare coding variants involving MYO7A and other genes encoding stereocilia link proteins in familial meniere disease. Hear. Res..

[91] Frejo L., Lopez-Escamez J.A. (2023). Recent advances in understanding molecular bases of Ménière’s disease. Fac. Rev..

[92] Gallego-Martinez A., Escalera-Balsera A., Trpchevska N., Robles-Bolivar P., Roman-Naranjo P., Frejo L., Perez-Carpena P., Bulla J., Gallus S., Canlon B. (2022). Using coding and non-coding rare variants to target candidate genes in patients with severe tinnitus. NPJ Genom. Med..

[93] Skarp S., Korvala J., Kotimäki J., Sorri M., Männikkö M., Hietikko E. (2022). New Genetic Variants in CYP2B6 and SLC6A Support the Role of Oxidative Stress in Familial Ménière’s Disease. Genes.

[94] Manche S.K., Jangala M., Putta P., Koralla R.M., Akka J. (2016). Association of oxidative stress gene polymorphisms with presbycusis. Gene.

[95] Altissimi G., Colizza A., Cianfrone G., de Vincentiis M., Greco A., Taurone S., Musacchio A., Ciofalo A., Turchetta R., Angeletti D. (2020). Drugs inducing hearing loss, tinnitus, dizziness and vertigo: An updated guide. Eur. Rev. Med. Pharmacol. Sci..

[96] Wang C., Zou Q., Pu Y., Cai Z., Tang Y. (2023). Berberine Rescues D-Ribose-Induced Alzheimer’s Pathology via Promoting Mitophagy. Int. J. Mol. Sci..

[97] Babolmorad G., Latif A., Domingo I.K., Pollock N.M., Delyea C., Rieger A.M., Allison W.T., Bhavsar A.P. (2021). Toll-like receptor 4 is activated by platinum and contributes to cisplatin-induced ototoxicity. EMBO Rep..

[98] Gong T., Liu L., Jiang W., Zhou R. (2020). DAMP-sensing receptors in sterile inflammation and inflammatory diseases. Nat. Rev. Immunol..

[99] Liu T., Zhang L., Joo D., Sun S.C. (2017). NF-κB signaling in inflammation. Signal Transduct. Target. Ther..

[100] Böttger E.C., Schacht J. (2013). The mitochondrion: A perpetrator of acquired hearing loss. Hear. Res..

[101] Podratz J.L., Knight A.M., Ta L.E., Staff N.P., Gass J.M., Genelin K., Schlattau A., Lathroum L., Windebank A.J. (2011). Cisplatin induced mitochondrial DNA damage in dorsal root ganglion neurons. Neurobiol. Dis..

[102] Xie J., Talaska A.E., Schacht J. (2011). New developments in aminoglycoside therapy and ototoxicity. Hear. Res..

[103] Fu X., Wan P., Li P., Wang J., Guo S., Zhang Y., An Y., Ye C., Liu Z., Gao J. (2021). Mechanism and Prevention of Ototoxicity Induced by Aminoglycosides. Front. Cell. Neurosci..

[104] Li N., Yan X., Huang W., Chu M., Dong Y., Song H., Peng Y., Shi J., Liu Q. (2023). Curcumin protects against the age-related hearing loss by attenuating apoptosis and senescence via activating Nrf2 signaling in cochlear hair cells. Biochem. Pharmacol..

[105] Marino G., Fernandez A.F., Cabrera S., Lundberg Y.W., Cabanillas R., Rodriguez F., Salvador-Montoliu N., Vega J.A., Germanà A., Fueyo A. (2010). Autophagy is essential for mouse sense of balance. J. Clin. Investig..

[106] Pak J.H., Kim Y., Yi J., Chung J.W. (2020). Antioxidant Therapy against Oxidative Damage of the Inner Ear: Protection and Preconditioning. Antioxidants.

[107] Cicero A.F., Baggioni A. (2016). Berberine and Its Role in Chronic Disease. Adv. Exp. Med. Biol..

[108] Feng X., Sureda A., Jafari S., Memariani Z., Tewari D., Annunziata G., Barrea L., Hassan S.T.S., Šmejkal K., Malaník M. (2019). Berberine in Cardiovascular and Metabolic Diseases: From Mechanisms to Therapeutics. Theranostics.

[109] Hou Q., He W.J., Wu Y.S., Hao H.J., Xie X.Y., Fu X.B. (2020). Berberine: A Traditional Natural Product with Novel Biological Activities. Altern. Ther. Health Med..

[110] Kim Y.R., Baek J.I., Lee K.Y., Kim U.K. (2023). Berberine chloride protects cochlear hair cells from aminoglycoside-induced ototoxicity by reducing the accumulation of mitochondrial reactive oxygen species. Free Radic. Biol. Med..

[111] Kilic K., Sakat M.S., Sahin A., Yildirim S., Dortbudak M.B. (2022). The effectiveness of berberine on noise-induced hearing loss: A rat model. Rev. Assoc. Med. Bras..

[112] Zhao Z., Han Z., Naveena K., Lei G., Qiu S., Li X., Li T., Shi X., Zhuang W., Li Y. (2021). ROS-Responsive Nanoparticle as a Berberine Carrier for OHC-Targeted Therapy of Noise-Induced Hearing Loss. ACS Appl. Mater. Interfaces.

[113] Fang X., Wu H., Wei J., Miao R., Zhang Y., Tian J. (2022). Research progress on the pharmacological effects of berberine targeting mitochondria. Front. Endocrinol..

[114] Hori I., Harashima H., Yamada Y. (2023). Development of a Mitochondrial Targeting Lipid Nanoparticle Encapsulating Berberine. Int. J. Mol. Sci..

[115] Qin X., Jiang M., Zhao Y., Gong J., Su H., Yuan F., Fang K., Yuan X., Yu X., Dong H. (2020). Berberine protects against diabetic kidney disease via promoting PGC-1α-regulated mitochondrial energy homeostasis. Br. J. Pharmacol..

[116] Kotha R.R., Luthria D.L. (2019). Curcumin: Biological, Pharmaceutical, Nutraceutical, and Analytical Aspects. Molecules.

[117] Bucak A., Ozdemir C., Ulu S., Gonul Y., Aycicek A., Uysal M., Cangal A. (2015). Investigation of protective role of curcumin against paclitaxel-induced inner ear damage in rats. Laryngoscope.

[118] Soyalıç H., Gevrek F., Koç S., Avcu M., Metin M., Aladağ İ. (2016). Intraperitoneal curcumin and vitamin E combination for the treatment of cisplatin-induced ototoxicity in rats. Int. J. Pediatr. Otorhinolaryngol..

[119] Abd-Elhakim Y.M., Abdel-Mota S.M., Malhat S.M., Mostafa H.I., Ibrahim W.M., Beheiry R.R., Moselhy A.A.A., Said E.N. (2022). Curcumin attenuates gentamicin and sodium salicylate ototoxic effects by modulating the nuclear factor-kappaB and apoptotic pathways in rats. Environ. Sci. Pollut. Res. Int..

[120] Yamaguchi T., Yoneyama M., Onaka Y., Imaizumi A., Ogita K. (2017). Preventive effect of curcumin and its highly bioavailable preparation on hearing loss induced by single or repeated exposure to noise: A comparative and mechanistic study. J. Pharmacol. Sci..

[121] So H., Kim H., Kim Y., Kim E., Pae H.O., Chung H.T., Kim H.J., Kwon K.B., Lee K.M., Lee H.Y. (2008). Evidence that cisplatin-induced auditory damage is attenuated by downregulation of pro-inflammatory cytokines via Nrf2/HO-1. J. Assoc. Res. Otolaryngol..

[122] Hu Y., Luo Y., Zheng Y. (2022). Nrf2 Pathway and Autophagy Crosstalk: New Insights into Therapeutic Strategies for Ischemic Cerebral Vascular Diseases. Antioxidants.

[123] Liao D., Shangguan D., Wu Y., Chen Y., Liu N., Tang J., Yao D., Shi Y. (2023). Curcumin protects against doxorubicin induced oxidative stress by regulating the Keap1-Nrf2-ARE and autophagy signaling pathways. Psychopharmacology.

[124] Han J., Pan X.Y., Xu Y., Xiao Y., An Y., Tie L., Pan Y., Li X.J. (2012). Curcumin induces autophagy to protect vascular endothelial cell survival from oxidative stress damage. Autophagy.

[125] Yu T., Wang L., Zhang L., Deuster P.A. (2023). Mitochondrial Fission as a Therapeutic Target for Metabolic Diseases: Insights into Antioxidant Strategies. Antioxidants.

